# Efficacy of novel agents against cellular models of familial platelet disorder with myeloid malignancy (FPD-MM)

**DOI:** 10.1038/s41408-024-00981-4

**Published:** 2024-02-05

**Authors:** Christopher P. Mill, Warren C. Fiskus, Courtney D. DiNardo, Patrick Reville, John A. Davis, Christine E. Birdwell, Kaberi Das, Hanxi Hou, Koichi Takahashi, Lauren Flores, Xinjia Ruan, Xiaoping Su, Sanam Loghavi, Joseph D. Khoury, Kapil N. Bhalla

**Affiliations:** 1 The University of Texas M. D. Anderson Cancer Center, Houston, TX 77030 USA; 2 University of Nebraska Medical Center, Omaha, NE 68198 USA

**Keywords:** Targeted therapies, Acute myeloid leukaemia

## Abstract

Germline, mono-allelic mutations in RUNX1 cause familial platelet disorder (RUNX1-FPD) that evolves into myeloid malignancy (FPD-MM): MDS or AML. FPD-MM commonly harbors co-mutations in the second RUNX1 allele and/or other epigenetic regulators. Here we utilized patient-derived (PD) FPD-MM cells and established the first FPD-MM AML cell line (GMR-AML1). GMR-AML1 cells exhibited active super-enhancers of MYB, MYC, BCL2 and CDK6, augmented expressions of c-Myc, c-Myb, EVI1 and PLK1 and surface markers of AML stem cells. In longitudinally studied bone marrow cells from a patient at FPD-MM vs RUNX1-FPD state, we confirmed increased chromatin accessibility and mRNA expressions of MYB, MECOM and BCL2 in FPD-MM cells. GMR-AML1 and PD FPD-MM cells were sensitive to homoharringtonine (HHT or omacetaxine) or mebendazole-induced lethality, associated with repression of c-Myc, EVI1, PLK1, CDK6 and MCL1. Co-treatment with MB and the PLK1 inhibitor volasertib exerted synergistic in vitro lethality in GMR-AML1 cells. In luciferase-expressing GMR-AML1 xenograft model, MB, omacetaxine or volasertib monotherapy, or co-treatment with MB and volasertib, significantly reduced AML burden and improved survival in the immune-depleted mice. These findings highlight the molecular features of FPD-MM progression and demonstrate HHT, MB and/or volasertib as effective agents against cellular models of FPD-MM.

## Introduction

RUNX1 is a DNA-binding subunit of the core binding factor (CBF) complex and master transcriptional regulator involved in normal and malignant hematopoiesis [1–3]. Most mono-allelic germline mutations in RUNX1 are missense or missense/truncation mutations that lead to nonsense mediated decay and loss of mutant (mt) RUNX1 protein, thereby behaving mostly as loss of function mutations [2–4]. Germline mutations in RUNX1 cause autosomal dominant Familial Platelet Disorder (FPD), with a propensity to evolve into myeloid malignancy (FPD-MM), i.e., MDS or AML [1–3, 5, 6]. FPD-MM harbors co-mutations, most commonly on the second allele of RUNX1, and on BCOR, PHF6, K/N-RAS, WT1 or TET2, which confer relative resistance to standard therapy for MDS or AML [2, 5, 7, 8]. Lack of specific targeted therapy and resistance to standard AML therapies accounts for poorer prognosis and outcome observed in FPD-MM [2, 7–9]. Although curative in some patients with FPD-MM, allogeneic stem cell transplantation from an unrelated, matched donor carries the risk of graft versus host disease and AML relapse [10, 11]. Therefore, there is a strong rationale and need to develop and test novel and effective small molecule drugs for FPD/MM, with the goal of eliminating the FPD-MM clone and reverting the disease back to RUNX1-FPD state. To achieve this goal, it is also important to develop the relevant in vitro cellular and patient-derived (PD) xenograft models of RUNX1-FPD and FPD-MM, which can be utilized for testing novel targeted therapies for FPD-MM.

In present studies we assessed the active enhancers and gene-expression alterations in PD bone marrow aspirate (BMA) cells harvested longitudinally during RUNX1-FPD and after it had evolved to FPD-MM. In a previous report, utilizing mRNA signature of RUNX1 knockdown by shRNA in AML cells harboring mtRUNX1, we had conducted LINCS (Library of Integrated Network-based Cellular Signatures) 1000-CMap (Connectivity Mapping) analysis [12, 13]. From this, we had identified expression mimickers (EMs), including the protein synthesis inhibitor homoharringtonine (HHT or omacetaxine) and anthelmintic fenbendazole (analog of mebendazole) [14–16]. Present studies determined greater lethal activity of HHT or mebendazole (MB) in PD cells from FPD-MM compared to RUNX1-FPD or normal CD34+ progenitor cells. From a patient who progressed from RUNX1-FPD (expressing mtRUNX1 K194N) to FPD-MM, with co-mutations documented by NGS in BCOR, PHF6, SF3B1 and SRSF2, we procured BMA cells and successfully established the first, continuously cultured cell line (GMR-AML1) expressing the same germline mtRUNX1. Findings presented here also highlight the molecular/genetic features associated with progression of RUNX1-FPD to FPD-MM, including those in the newly established GMR-AML1 cell line. Tail vein infusion and engraftment of luciferase-transduced GMR-AML1 caused splenomegaly and 100% mortality of NSG mice by approximately day-40, post-infusion. Treatment with HHT or mebendazole, versus vehicle control, significantly reduced GMR-AML1 burden and improved overall survival of NSG mice engrafted with GMR-AML1 cells. They also demonstrate that co-treatment with HHT and venetoclax, or MB with the polo-like kinase (PLK1) inhibitor volasertib [17], exerted synergistic lethality in GMR-AML1 cells. If these EMs exert greater lethality against HPCs from patients with FPD-MM compared to RUNX1-FPD, they will have the potential to revert FPD-MM back to RUNX1-FPD hematopoiesis.

## Materials and methods

### Primary FPD and FPD-MM samples

Patient-derived FPD and FPD-MM samples (peripheral blood and BMA) for the conduct of preclinical studies and for the creation of a cell line were obtained from patients with informed consent, approved by the MD Anderson Cancer Center’s Institutional Review Board (IRB# PA14-0392).

### Generation of a germline mutant Runx1 cell line (GMR-AML1)

The cell suspension of the original patient cells was adjusted to a concentration of 2 × 10^6^/mL in RPMI-1640 media with 20% FBS, 1% Pen-Strep, and 1% non-essential amino acids. Cells were incubated in a humidified incubator at 37 °C and 5% CO_2_ in air. Media was changed once per week until cells began to proliferate and grow in small floating clusters. When cells began to proliferate (less than two months in culture) and change the color of the media more frequently, media was changed twice per week by dilution or by centrifugation at 200 × *g* for 5 min. Early passages of the cells were cryopreserved in 90% FBS + 10% DMSO in 5–10 million cell aliquots and stored in liquid nitrogen to allow for characterization of the cell line and to monitor for genetic drift. The presence of germline Runx1 mutation was confirmed via Sanger sequencing.

### Whole exome analysis of GMR-AML1

Whole exome analysis was performed on GMR-AML1 cells utilizing Agilent Exome 7 (SureSelect Human All Exon v7). The raw paired-end (PE) reads in FASTQ format was aligned to the human reference genome (hg38) for human DNA-Seq, using BWA alignment software. GMR-AML1 mutant calls were cataloged and are reported.

### Cell lines and cell culture

OCI-AML5 [DSMZ Cat# ACC-247, RRID:CVCL_1620] and OCI-AML2 [DSMZ Cat# ACC-99, RRID:CVCL_1619] cells were obtained from the DSMZ. HEK-293T cells were obtained from the Characterized Cell Line Core Facility at M.D. Anderson Cancer Center, Houston TX. All experiments with cell lines were performed within 6 months after thawing or obtaining from DSMZ. The cell lines were also authenticated in the Characterized Cell Line Core Facility at M.D. Anderson Cancer Center, Houston TX. OCI-AML2 and OCI-AML5 were cultured in ribonucleoside-containing Alpha-MEM media with 20% FBS, 1% non-essential amino acids (NEAA), and 1% penicillin/streptomycin. OCI-AML5 cells were supplemented with 10 µg/mL concentration of GM-CSF. HEK-293T cells were cultured in high-glucose-formulated DMEM media with 10% FBS, 1% NEAA, 1% L-glutamine, and 1% penicillin/streptomycin. Logarithmically growing, mycoplasma-negative cells were utilized for all experiments. Following drug treatments, cells were washed free of the drug(s) prior to the performance of the studies described.

### Cell line authentication

The cell lines utilized in these studies were authenticated in the Characterized Cell Line Core Facility at M.D. Anderson Cancer Center, Houston TX utilizing STR profiling.

### Statistical analysis

Significant differences between values obtained in AML cells treated with different experimental conditions compared to untreated control cells were determined using the Student’s *t* test in GraphPad V9. For the in vivo mouse models, a two-tailed, unpaired *t* test was utilized for comparing total bioluminescent flux. For survival analysis, a Kaplan–Meier plot and a Mantel–Cox log rank test were utilized for comparisons of different cohorts. *P* values of <0.05 were assigned significance.

## Results

### Establishing the first FPD-MM GMR-AML cell line

**Fig. 1:**
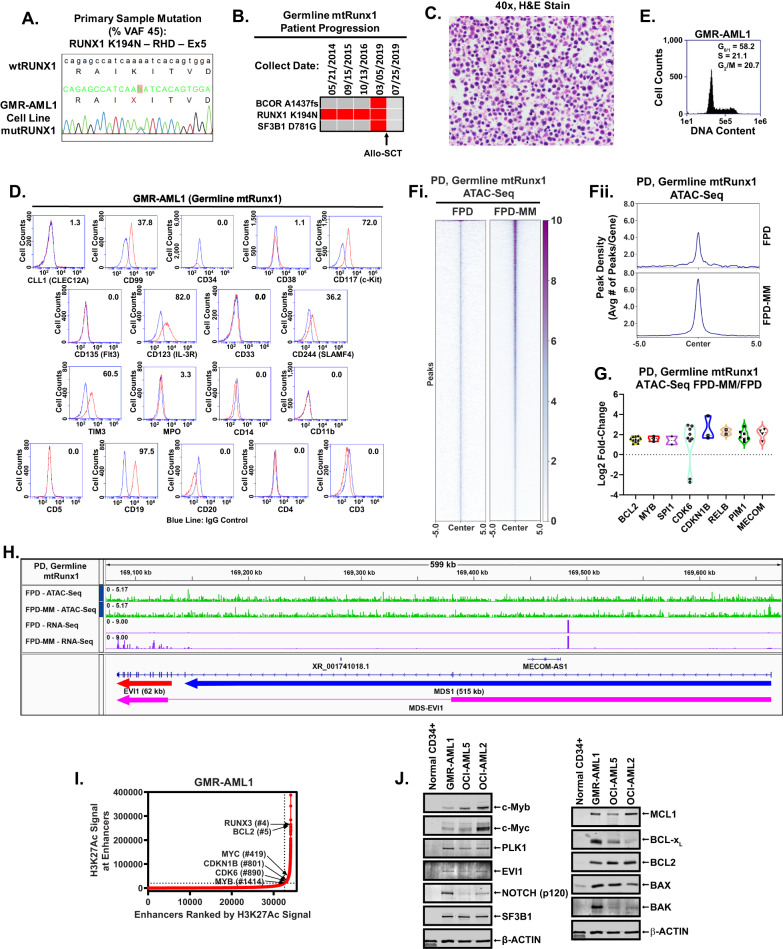
Characterization of a germline mutant Runx1 AML (GMR-AML1) cell line. **A** Electropherogram of the Runx1 mutation identified in GMR-AML1. **B** Oncoplot of mutations identified by Next-Generation Sequencing (NGS) during progression of a germline mutant Runx1 patient. **C** H & E stained cells from a representative Formalin-Fixed Paraffin-Embedded (FFPE) section showing the morphology of GMR-AML1 cells. **D** Flow cytometry analysis of AML relevant myeloid, progenitor, and B/T cell markers. **E** GMR-AML1 cells in exponential phase were harvested and fixed for cell cycle analysis. Panel shows representative DNA content/cell cycle status as determined by flow cytometry. **F** Global ATAC-Seq heat map and peak profile in longitudinal FPD-MM vs. FPD at peak +/− 5 kb resolution. **G** The log2 fold-increase (>1.25-fold) in accessibility of genes in FPD-MM vs. FPD as identified by ATAC-Seq analysis. **H** IGV plot showing peak density of Tn5-accessible chromatin and RNA-Seq peak profile in primary, patient-derived FPD-MM and FPD samples. **I** GMR-AML1 cells in exponential phase were crosslinked with formaldehyde. H3K27Ac ChIP-Seq analysis was conducted on sonicated chromatin. Ranked ordering of super enhancers (ROSE) analysis was conducted. Rank of individual super enhancers are shown relative to the total number of super enhancers. **J** Baseline protein expressions in GMR-AML1 cells compared to normal HPCs or leukemia cell lines OCI-AML5 (somatic mtRunx1) and OCI-AML2 (wtRunx1).

**Fig. 2:**
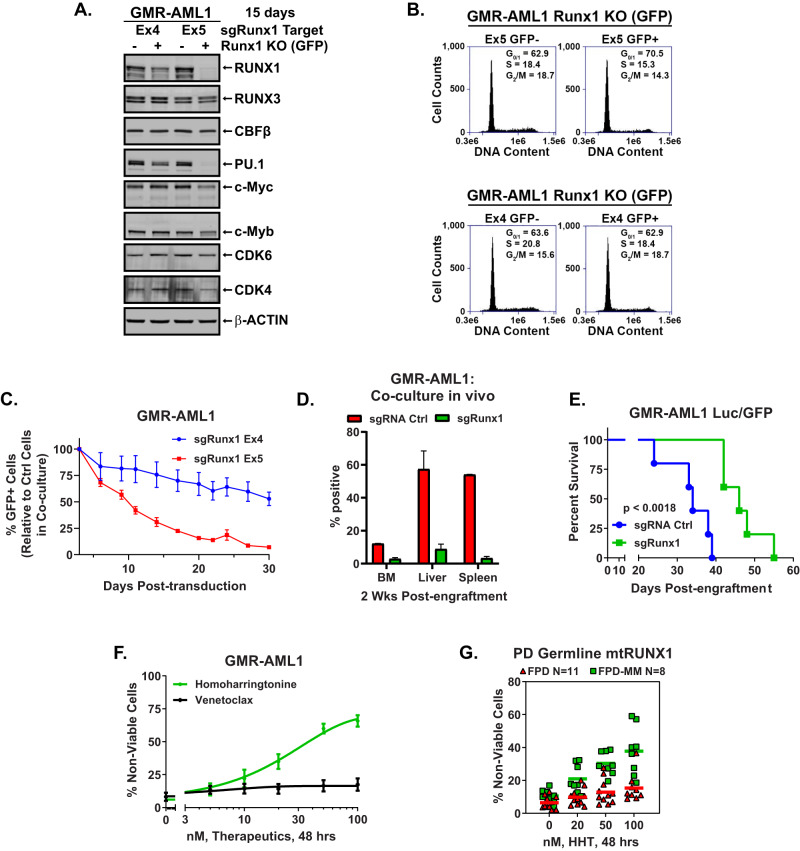
Guide RNA-mediated knockout of Runx1 depletes c-Myc, PU.1, c-Myb, and CDK4, induces cell cycle G0/G1 arrest, and depletes competitive advantage in GMR-AML1 cells. **A** GMR-AML1/Cas9 cells were transduced with lentivirus expressing Runx1 guide RNAs and eGFP for 72 h. Cells were sorted by FACS into GFP positive and negative populations and cultured for an additional 12 days. Following this immunoblot analyses were conducted on total cell lysates. The expression of β-Actin in the lysates served as the loading control. **B** GMR-AML1/Cas9 cells transduced and cultured as in (**A**). were harvested and fixed for cell cycle analysis. Panel shows representative DNA content/cell cycle status as determined by flow cytometry. **C** GMR-AML1/Cas9 cells transduced as in (**A**). were mixed (+/− GFP) and co-cultured for 30 days. The percent GFP+ cells were measured every 3–4 days by flow cytometry. The line represents the mean percent of GFP+ cells from four experiments +/− S.E.M. **D** GMR-AML1/Cas9 cells transduced as in (**A**). with sgRNA Ctrl (tdTomato) or sgRunx1 Ex5 (eGFP) were cultured for seven days then mixed at a 1:1 ratio and tail vein injected into pre-irradiated NSG mice. Mice were monitored daily for two weeks and then two mice were euthanized. Bone marrow, liver, and spleen were harvested and analyzed for tdTomato and GFP positive cells by flow cytometry. **E** GMR-AML1/Cas9 cells were transduced and cultured as in (**D**). and separately injected into NSG mice (*N* = 5 per cohort). The Kaplan-Meier survival curve shows the median and overall survival of NSG mice bearing GMR-AML1/Cas9 cells transduced with sgRNA control or sgRunx1. Significance was determined by a Mantel-Cox log rank test. **F** GMR-AML1 cells were treated with the indicated concentrations of HHT or venetoclax for 48 h. Then the percentage non-viable cells were determined by TO-PRO-3 iodide staining and flow cytometry. **G** PD germline mutant Runx1 FPD and FPD-MM cells were treated ex vivo with the indicated concentrations of HHT for 48 h. Then the percentage non-viable cells were determined by TO-PRO-3 iodide staining and flow cytometry.

To address the need to develop relevant in vitro and in vivo models and test novel targeted therapies for FPD-MM, we successfully established the first, continuously cultured cell line (GMR-AML1) expressing germline mtRUNX1. This cell line was generated from the BMA cells from a patient with FPD-MM that developed from RUNX1-FPD expressing mtRUNX1 K194N (Fig. 1A). In this patient, progression to FPD-MM was associated with co-mutations, including BCOR A1437fs (VAF 13%) and SF3B1 D781G (VAF 4%), as documented by NGS (Fig. 1B). Notably, the mutations identified by NGS in the FPD-MM cells from the patient were not detected by NGS in the GMR-AML1 cell line except for the RUNX1 K194N, which was also identified in other pedigree members who had developed FPD (Fig. S1A). Additionally, one of the members of the pedigree also exhibited transformation of FPD to FPD-MM. This attests to the biologic significance of the presence of the germline RUNX1 K194N mutation. GMR-AML1 cells were cytogenetically diploid and lacked MYC or MLL1 rearrangement, or other copy number gains or losses on array CGH (Fig. S1B, C and not shown). Instead, whole exome sequencing (WES) performed on GMR-AML1 cell line identified additional mutations in TP53 (P72R), AIM2 (K340fs), NELFB (L523F), CEP152 (Y370X), SUGP2 (H23L), RRM2B (R71fs), TADA3 (T27R), SPDYE6 (G292C) and PRDM9 (S814R) with % VAF ranging between 33 to 55% (Fig. S1D). The functional significance of TP53 codon 72 alteration for the transformation to FPD-MM is unclear [18]. Collectively, these genetic alterations suggest that GMR-AML1 cell line is derived from the clonal expansion under in vitro culture conditions of AML stem-progenitor cells present in the BMA of the patient with FPD-MM. The in vitro survival and growth of GMR-AML1 cells is likely promoted by these WES-detected mutations. We next further characterized the biologic features of GMR-AML1 cell line expressing mtRUNX1. Figure 1C shows the morphologic features of the GMR-AML1 cells. Most of these cells are poorly differentiated myeloid progenitors. As determined by flow cytometry, GMR-AML1 cells express CD117, CD123, CD99, CD33, TIM3, CD86, and CD18, but not CD34, CLEC12A, CD38, CD244, CD11b, CD14, CD3 and CD5 (Fig. 1D). Flow cytometry also showed the cell cycle distribution of GMR-AML1 cells, with 58.2, 21.1 and 20.7% of cells in G1, S and G2/M phase of the cell cycle, respectively (Fig. 1E). Additionally, tail vein-infused GMR-AML1 cells in NSG mice engrafted in the mice, resulting in AML growth and mortality of all mice in 4 to 5 weeks post engraftment (vide infra, Fig. 2E).

### Alterations in chromatin accessibility and gene expressions in FPD-MM and GMR-AML1 cells

We next conducted ATAC-Seq and RNA-Seq analyses on cells harvested longitudinally from a patient at the RUNX1-FPD versus FPD-MM stage [19]. Figure 1F demonstrates that, compared to RUNX1-FPD, FPD-MM cells exhibited a significant increase in chromatin accessibility. Notably, log2-fold increase in ATAC-Seq peaks was observed at several loci, including MECOM, PIM1, RELB, CDKN1B, CDK6, SPI1, MYB and BCL2 loci, whereas ATAC-Seq peaks declined at the PRDM9, WNT5A, TERT and AF3 loci (Figs. 1G and S1E). RNA-Seq analysis showed significant increase in 404 mRNA expressions and decline in 1305 mRNA expressions in FPD-MM cells, as compared to RUNX1-FPD cells (Fig. S1F). QPCR analysis showed significant increase in BCL2 and MYB but decline in RUNX1 and SPI1 mRNA expressions (Fig. S1G). Notably, ATAC-Seq and RNA-Seq peaks significantly increased at the MECOM locus, involved in AML stem cell renewal and differentiation arrest [20], in FPD-MM compared to RUNX1-FPD cells (Fig. 1H). We next conducted ChIP analysis with anti-H3K27Ac antibody in GMR-AML cells [4, 19]. This demonstrated high occupancy of H3K27Ac at the active super-enhancers of 1478 genes, including those of MYC, RUNX3, MYB, CDKN1B, BCL2 and CDK6 (Fig. 1I) [21]. To assess gene expressions, we performed immunoblot analyses to compare protein expressions in GMR-AML1 cells with germline mtRUNX1 versus OCI-AML5 and OCIAML2 cells, which express somatic heterozygous mtRUNX1 and two copies of wtRUNX1 [12], respectively, as well as versus CD34+ normal hematopoietic progenitor cells. Figure 1J demonstrates that, compared to normal CD34+ progenitor cells, GMR-AML1 cells expressed higher levels of c-Myb, EVI1, c-Myc, polo-like kinase 1 (PLK1), NOTCH1, BCL2, BcL-xL, MCL1, BAX and BAK. However, compared to OCI-AML5, GMR-AML1 cells expressed lower levels of c-Myb, c-Myc and BCL2 (Fig. 1J). These findings highlight the increased activity of chromatin and increased expression of specific oncoproteins that are associated with arrested differentiation, increased in vitro growth and in vivo leukemogenic potential of GMR-AML1 cells.

### Effect of RUNX1 depletion on cell growth and sensitivity to homoharringtonine (HHT, omacetaxine) in GMR-AML1 and FPD-MM cells

We next determined the effects of RUNX1 depletion by CRISPR knock out (KO) on cell growth and drug sensitivity of GMR-AML1 cells. These cells, transduced with and expressing Cas9, were further transduced with a lentivirus expressing a RUNX1 gRNA (targeting either exon 4 or 5) and EGFP. Forty-eight hours after transduction, the cells were sorted into GFP positive (Runx1 KO) or negative control (Ctrl) populations. Immunoblot analysis showed that, compared to the exon 4 gRNA, exon 5 gRNA-mediated RUNX1 KO led to greater depletion of RUNX1, PU.1, c-Myc, c-Myb, CDK4/6, but not of RUNX3 or CBFβ in GMR-AML1 cells (Fig. 2A). Compared to the control, exon 5 gRNA more than exon 4 gRNA, increased the % of G1 and reduced the % of cells in S and G2/M phases of the cell cycle, likely due to greater decline in the levels of the cell cycle regulatory oncoproteins (Fig. 2B). Next, the same number of GFP positive (RUNX1 KO) and negative (Ctrl) GMR-AML1 cells were mixed and co-cultured for 30 days. The percentage of GFP-positive cells was determined via flow cytometry, following passage of the cells every 3 days in culture. In this competitive in vitro culture assay, depletion of RUNX1 via RUNX1-KO was associated with marked decline in the GFP + GMR-AML1 compared to the control cells (Fig. 2C). Compared to sgRNA control, RUNX1 knockout in GMR-AML1 cells also reduced engraftment potential in bone marrow, liver, and spleen of NSG mice during an in vivo competition assay (Fig. 2D and S2A). Additionally, NSG mice engrafted with GMR-AML1 cells in which RUNX1 was knocked out, compared to sgRNA control-treated cells engrafted in the mice, exhibited significantly delayed progression and prolonged survival (Fig. 2E).

We had previously reported that, compared to AML cells expressing two copies of wtRUNX1, in the isogenic AML cells expressing heterozygous mtRUNX1, RUNX1 depletion caused greater sensitivity to homoharringtonine (HHT or omacetaxine)-induced cell death, associated with reduced levels of c-Myc, c-Myb, MCL1 and Bcl-xL [12]. In present studies also, exposure to HHT for 48 h dose-dependently induced loss of viability in GMR-AML1 cells, whereas treatment with venetoclax was ineffective (Fig. 2F), likely due to relatively high expression of MCL1, Bcl-xL and BFL-1 (Fig. 1J and vide infra) [22, 23]. Additionally, treatment with daunorubicin, panobinostat (a pan-HDAC inhibitor), cytarabine or etoposide also dose-dependently induced apoptosis in GMR-AML1 cells (Fig. S2B) [24]. However, GMR-AML1 cells were insensitive to A1155463 (Bcl-xL inhibitor), AZD5991 (MCL1 inhibitor), the DNA hypomethylating drugs, azacytidine and decitabine, or INCB059872 (LSD1 inhibitor inhibitor)-induced loss of viability (Fig. S2B) [25]. In addition to GMR-AML1 cells, we also determined the dose dependent-lethal effects of HHT on the BMA cells from 11 patients with FPD versus 8 patients with FPD-MM (Fig. 2G). As shown in Fig. 2G, HHT exerted greater lethal activity against FPD-MM as compared to FPD cells. Fig. S2C, D show the oncoplot of the co-mutations and specific RUNX1 mutations in each of the BMA sample cells from FPD versus FPD-MM patients, respectively.

### Effects of HHT on chromatin accessibility and gene expressions in GMR-AML and FPD-MM cells

**Fig. 3:**
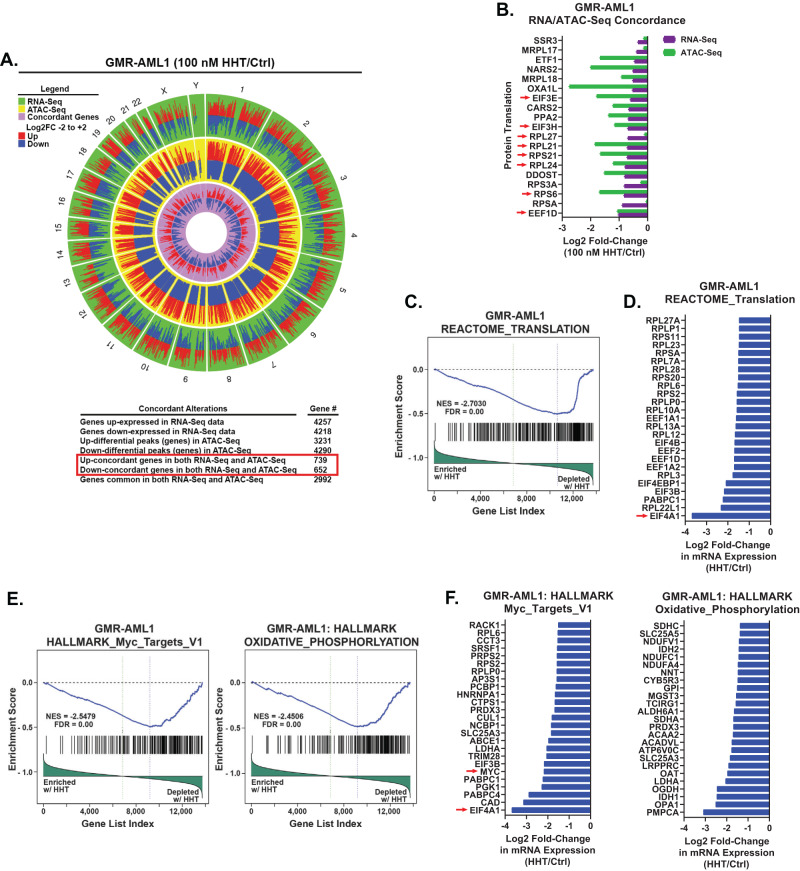
Treatment with HHT induces concordant ATAC and RNA-Seq expression alterations in GMR-AML1 cells. **A**, **B** GMR-AML1 cells were treated with 100 nM of HHT for eight hours and ATAC-Seq and RNA-Seq analyses were performed. A Circos plot and the log2 fold-change of selected concordant ATAC-Seq and mRNA expression alterations in HHT-treated GMR-AML1 cells are shown. **C**–**F** Gene set enrichment analysis (GSEA) and selected target fold changes in HHT-treated GMR-AML1 cells compared with HALLMARK and REACTOME pathway datasets.

Utilizing bulk RNA-Seq and ATAC-Seq analyses, we compared the chromatin accessibility and mRNA gene-expression perturbations in HHT treated (100 nM for 8 h) versus the untreated control GMR-AML1 cells. Genome wide, 739 genes showed significant and concordant increase in ATAC-Seq and RNA-Seq peaks, whereas 652 genes showed a concordant decline in the peaks [26], prominently among these were the ribosomal genes involved in protein translation (Fig. 3A, B). ATAC-Seq and RNA-Seq peaks also concordantly declined at numerous RNA-Pol II transcribed genes, including DNA POLR2B, TBL1X, CHD4, IGFBP3, CCNE1, CCND2, HDAC5/7, PTPN11, Caspase-8 and RRM2 (Fig. S3). RNA-Seq analysis of GMR-AML1 cells showed that the reactome of gene expressions involving protein translation was negatively enriched, with significant decline in the mRNA of EIF4A1 (Fig. 3C, D). HHT treatment also negatively enriched gene expressions in the gene-sets of MYC targets and oxidative phosphorylation in GMR-AML1 cells (Fig. 3E, F).

**Fig. 4:**
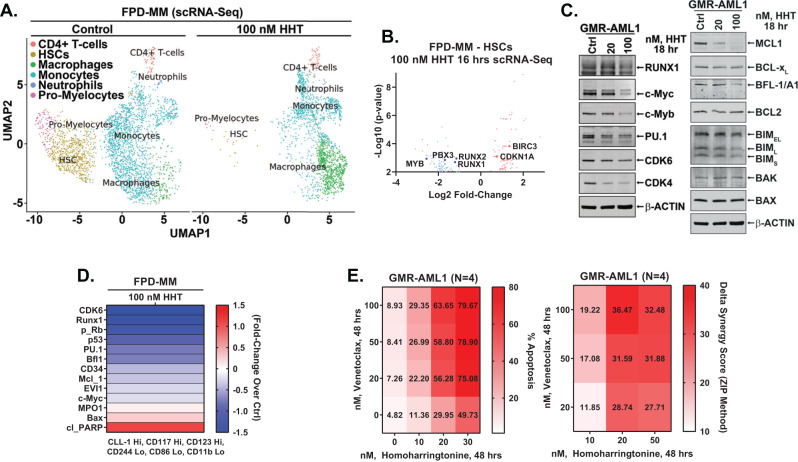
Treatment with HHT depletes the HSC population in BM-derived primary FPD-MM cells and exerts in vitro synergistic anti-leukemia activity. **A** Primary FPD-MM18 cells were treated with 100 nM of HHT for 16 h and single cell RNA-Seq (scRNA-Seq) analysis was performed. SingleR analysis was utilized to define cell clusters. The UMAP plot shows defined cell populations. **B** Log2 fold-change in mRNA expressions due to HHT treatment in the HSC population. **C** GMR-AML1 cells were treated with the indicated concentrations of HHT for 18 h. Following this, immunoblot analyses were conducted on total cell lysates. The expression of β-actin in the lysates served as the loading control. **D** Patient-derived FPD-MM18 cells were treated with 100 nM of HHT for 18 h. Cells were harvested and analyzed by CyTOF analysis utilized a cocktail of rare metal element-tagged antibodies. Leukemia stem cells were defined by high expression of CLEC12A(CLL-1), CD117, and CD123 and low expression of CD244, CD86, and CD11b. Heat map shows the absolute fold-change of significantly altered protein expressions in the treated over control cells. **E** GMR-AML1 cells were treated with the indicated concentrations of HHT and/or venetoclax for 48 h. Then the percentage annexin V-positive apoptotic cells was determined by flow cytometry. Delta synergy scores (ZIP) were determined using SynergyFinder v3.0 web application.

In addition to GMR-AML1 cells, utilizing scRNA-Seq analyses, we also determined the effects of HHT treatment on mRNA expressions in PD FPD-MM BMA cells [12, 26]. Figures 4A and S4A demonstrate that, following HHT treatment, there was a marked reduction in the HSC and pro-myelocyte populations of cells, whereas the cell numbers of macrophages increased markedly. In the residual HSC cluster of cells, there was log2-fold decline in MYB, PBX3 and RUNX1/2 mRNA expressions, but significant increase in mRNA of CDKN1A and BIRC3 (Fig. 4B). Bulk RNA-Seq performed on an FPD-MM sample also showed positive enrichment of the mRNA expressions belonging to the gene-sets (HALLMARK) of apoptosis signaling and TP53 targets but negative enrichment of c-Myc targets (Fig. S4B). Notably, following HHT treatment, there was also negative enrichment of the reactomes of G2/M checkpoint, DNA replication, cell-cycle checkpoints, E2F mediated regulation of DNA replication, mitotic spindle checkpoint and nuclear envelope breakdown (Fig. S4C). RNA-Seq analysis of FPD-MM cells also showed that HHT treatment caused log2-fold decline in mRNA of MYC, MYB, MCM2/4, FOXM1 and myeloperoxidase (MPO) but increase in CDKN1A, CDKN2B, BCL2A1, ATF3/4, PMAIP1 and HMOX1 (Fig. S4D). Notably, RNA-Seq evaluation of a separate PD FPD-MM cell sample revealed similar effects of HHT treatment on gene-expressions (Fig. S4E–J). We also compared baseline mRNA expressions and response to HHT in GMR-AML1 and the two FDP-MM samples. As shown in Fig. S4K, L, we observed marked similarity in gene expression at baseline and following HHT treatment in the two FPD-MM samples and GMR-AML1 cells. Following HHT treatment, immunoblot analyses showed that, compared to untreated controls, GMR-AML1 cells exhibited reduced protein levels of c-Myb, c-Myc, PU.1, CDK4/6, MCL1, BFL1, Bcl-xL and RUNX1 (Fig. 4C). Mass cytometry conducted on the same BMA cells showed that in the phenotypically characterized FPD-MM stem cells, based on high surface expression of CLL1 (CLEC12A), CD117, and CD123, but low expression of CD11b, CD244 and CD86, HHT treatment reduced the expression of CD34, c-Myc, EVI1, MCL1, Bfl1, p53, p-RB, PU.1, RUNX1 and CDK6 (Fig. 4D). The effects of HHT treatment on protein expressions also explain why, although GMR-AML1 cells were relatively insensitive to treatment with venetoclax, cotreatment with HHT and venetoclax synergistically induced apoptosis of GMR-AML1 cells (Fig. 4E).

### Lethal activity of mebendazole (MB), other anti-mitosis agents and established anti-AML agents in GMR-AML1 cells

**Fig. 5:**
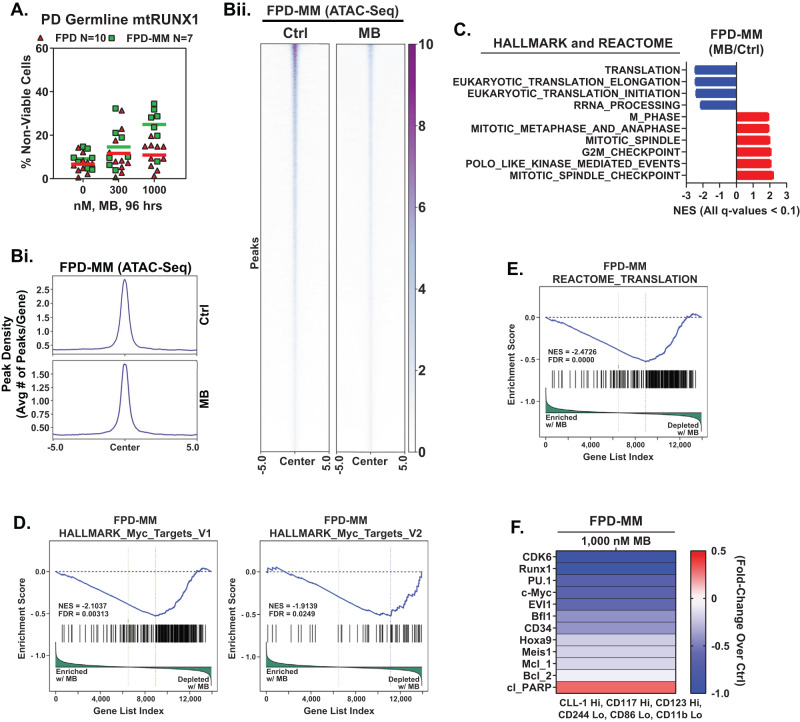
Treatment with MB reduces chromatin accessibility, negatively enriches mRNA expression in MYC targets and translation gene sets, reduces protein expression in PD, FPD-MM leukemia stem cells and induced greater loss of viability in FPD-MM compared to FPD. **A** PD germline mutant Runx1 FPD and FPD-MM cells were treated ex vivo with the indicated concentrations of MB for 96 h. Then, the percentage non-viable cells was determined by TOPRO-3 iodide staining and flow cytometry. **B** Global ATAC-Seq heat map and peak profile in PD, FPD-MM17 cells treated with 1000 nM MB for 24 h at peak +/− 5 kb resolution. **C**–**E** Primary FPD-MM18 cells were treated with 1000 nM of MB for 24 h and bulk RNA-Seq analysis was performed. Gene set enrichment analysis (GSEA) in MB-treated FPD-MM18 cells compared with HALLMARK and REACTOME pathway datasets. **F** Patient-derived FPD-MM18 cells were treated with 1000 nM of MB for 24 h. Cells were harvested and analyzed by CyTOF analysis utilized a cocktail of rare metal element-tagged antibodies. Leukemia stem cells were defined by high expression of CLL-1, CD117, and CD123 and low expression of CD244, CD86, and CD11b. Heat map shows the absolute fold-change of significantly altered protein expressions in the treated over control cells.

**Fig. 6:**
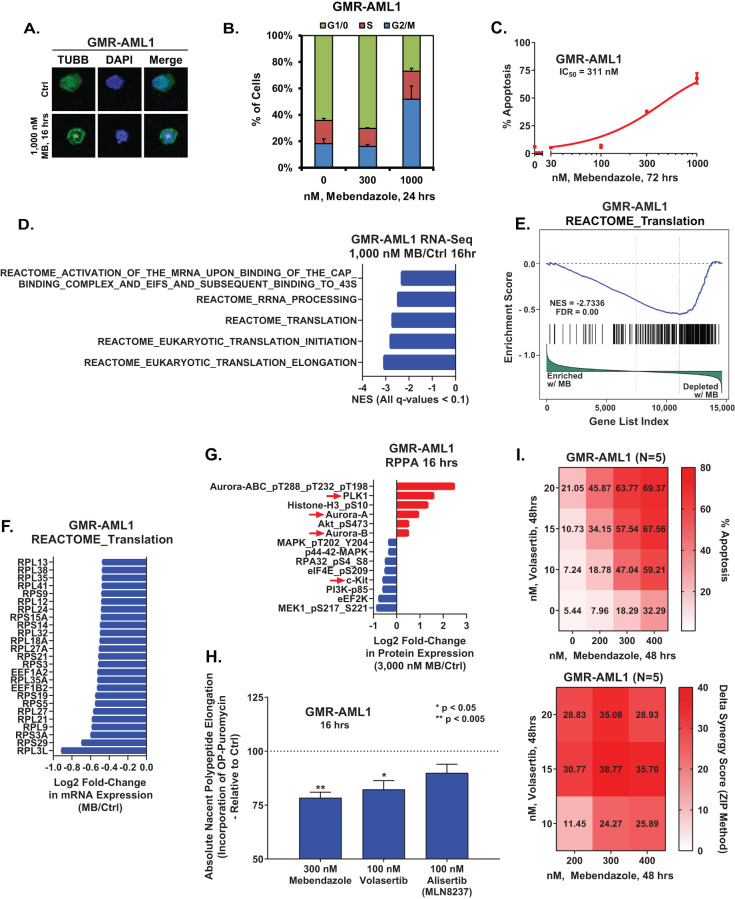
Treatment with MB augments Tubulin polymerization, induces G2/M arrest, negatively enriches translation pathways, inhibits nascent protein synthesis, and induces synergistic loss of viability with PLK1 inhibitor, Volasertib, in GMR-AML1 cells. **A** GMR-AML1 cells were treated with the indicated concentration of MB for 16 h. Cells were fixed, permeabilized, and stained with β-Tubulin antibody. DNA were stained with DAPI. Cells were imaged by confocal microscopy. Original magnification is 60×. **B** GMR-AML1 cells were treated with the indicated concentration of MB for 24 h. Cells were fixed with 70% molecular grade ethanol, stained with propidium iodide, and cell cycle analysis was determined by flow cytometry. **C** GMR-AML1 cells were treated with the indicated concentrations of MB for 72 h. Then the percentage apoptotic cells were determined by Annexin V and TO-PRO-3 iodide staining and flow cytometry. **D**–**F** GMR-AML1 cells were treated with 1000 nM of MB for 16 h and bulk RNA-Seq analysis was performed. Gene set enrichment analysis (GSEA) in MB treated cells compared with HALLMARK and REACTOME pathway datasets. **G** GMR-AML1 cells were treated with 3000 nM MB for 16 h in biologic triplicates. Reverse phase protein array (RPPA) analysis was conducted. Log2 fold-changes in selected significantly altered proteins are shown. **H** GMR-AML1 cells were treated with the indicated concentrations of MB, Volasertib, or Alisertib for 16 h. Nascent polypeptide elongation was detected by OPP puromycin incorporation assay and flow cytometry. Column represents mean of six independent experiments; bar represents SEM. **I** GMR-AML1 cells were treated with the indicated concentrations of MB and/or Volasertib for 48 h. Then the percentage annexin V-positive apoptotic cells were determined by flow cytometry. Delta synergy scores (by ZIP method) were determined using SynergyFinder v3.0.

Based on the EMs revealed by the LINCS1000 CMap analysis described in a previous report [4], we also determined the effects of mebendazole (MB) on freshly procured FPD-MM vs RUNX1-FPD cells. As shown in Fig. 5A, MB induced greater loss of viability in FPD-MM as compared to RUNX1-FPD cells. In contrast, MB treatment was markedly less toxic toward normal CD34+ HPCs (Fig. S5). We next determined the effects of MB treatment on chromatin accessibility and transcription in FPD-MM cells. Figure 5B demonstrates that exposure to MB significantly reduced genome wide chromatin accessibility, as determined by ATAC-Seq peaks density in FPD-MM cells. Following MB treatment of FPD-MM cells, RNA-Seq analysis revealed significant positive enrichment of the gene-sets involved in G2/M checkpoint, mitotic metaphase and anaphase, mitosis, spindle checkpoint, mitotic spindle and polo-like kinase mediated events (Fig. 5C). In contrast, there was also negative enrichment of the gene-sets of ribosomal RNA processing, eukaryotic translation initiation/elongation and protein translation (Fig. 5C). Specifically, HALLMARK gene-sets of c-Myc targets and the reactome of protein translation were negatively enriched (Fig. 5D, E). Guided by these MB-mediated mRNA perturbations, we conducted CyTOF analysis to determine the effects of MB treatment on specific protein expressions. As shown in Fig. 5F, MB treatment reduced the protein levels of RUNX1, CDK6, PU.1, c-Myc, EVI1, HOXA9, MEIS1, BFL1 and MCL1 in phenotypically-defined FPD-MM myeloid stem-progenitor cells [26, 27]. In GMR-AML1 cells, consistent with tubulin polymerization activity of MB, shown in Fig. 6A, MB treatment for 24 h induced increase in % of cells in the G2/M phase of the cell-cycle (Fig. 6B). Exposure to >300 nM of MB for 72 h also induced loss of viability in GMR-AML1 cells (Fig. 6C). RNA-Seq analysis conducted in GMR-AML1 cells following treatment with MB revealed negative enrichment of the reactome of ribosomal RNA processing, eukaryotic translation initiation/elongation and protein translation, as well as negative enrichment of the MYC targets (Figs. 6D, E, S6A, B). A log2-fold significant decline in the mRNA of ribosomal proteins was observed in the reactome of protein translation in GMR-AML1 cells (Fig. 6F). RPPA (reversed phase protein array) analysis of GMR-AML1 cells revealed that MB treatment increased protein expression of p-Aurora A/B/C kinases, Aurora A/B kinase and PLK1, but reduced protein levels of p-MEK, eEF2K, PI3K-p85, p-eIF4E and MAPK (Fig. 6G). Based on these perturbations due to MB treatment, we also determined the effects of treatment with MB, volasertib (PLK1 inhibitor) or alisertib (Aurora A kinase inhibitor) on nascent polypeptide elongation [12, 28]. Exposure to MB or volasertib, but not alisertib, significantly inhibited nascent polypeptide elongation in GMR-AML1 cells (Fig. 6H). Indeed, co-treatment with MB and volasertib or plogosertib induced synergistic lethality in GMR-AML1 cells, with delta synergy scores of >10 by the ZIP method (Figs. 6I and S6C). In contrast, treatment with volasertib did not induce significant lethality, or its co-treatment with MB did not significantly increase MB-induced lethality in CD34+ normal progenitor cells (Fig. S6D, E). These findings highlight the lethal activity and associated molecular perturbations due to MB and/or volasertib against cellular models of FPD-MM.

### In vivo efficacy of omacetaxine (HHT), MB and/or volasertib in a xenograft model of GMR-AML1 cells

**Fig. 7:**
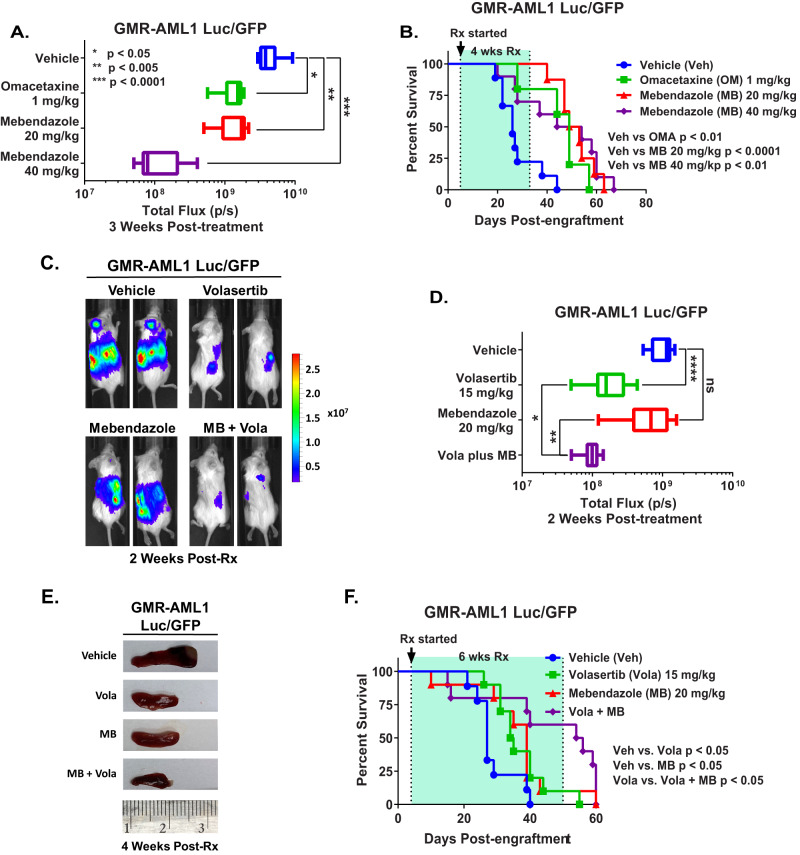
Treatment with Omacetaxine, Mebendazole, or Volasertib reduce leukemia burden and improve overall survival of NSG mice engrafted with GMR-AML1 cells. **A** Total bioluminescent flux (P/S) in NSG mice engrafted with (1 million) GMR-AML1 cells and treated for three weeks as indicated. **B** Kaplan–Meier survival curve of NSG mice engrafted with (1 million) GMR-AML1 cells and treated for four weeks as indicated. Significance was determined by a Mantel-Cox log rank test. **C** Representative images of mice engrafted with GMR-AML1 Luc/GFP cells and treated with 20 mg/kg MB and/or 15 mg/kg Volasertib for two weeks and IVIS imaged. **D** Total bioluminescent flux (P/S) in NSG mice engrafted with (1 million) GMR-AML1 cells and treated for two weeks as indicated. *p < 0.05; **p < 0.005; ****p < 0.0001. **E** Representative images of spleens from mice engrafted with GMR-AML1 Luc/GFP cells and treated with 20 mg/kg MB and/or 15 mg/kg Volasertib for four weeks. **F** Kaplan-Meier survival curve of NSG mice engrafted with (1 million) GMR-AML1 cells and treated for six weeks as indicated. Significance was determined by a Mantel-Cox log rank test.

We next determined the in vivo anti-leukemia efficacy of omacetaxine (HHT or OM) or mebendazole in the xenograft model of luciferase/GFP transduced GMR-AML1 cells. Following tail vein infusion and engraftment of GMR-AML1-Luc/GFP cells, cohorts of NSG mice were treated with vehicle control or previously determined safe doses of the drugs [12, 14]. Figure 7A demonstrates that following three weeks of treatment, single agent OM or MB, compared to vehicle control, significantly reduced leukemia burden due to GMR-AML1 cells, with greater reduction caused by treatment with 40 mg/kg of MB. Following 4-weeks of treatment, each drug compared to vehicle control also significantly improved survival of NSG mice, without inducing toxicity (Fig. 7B). In a separate study, cohorts of mice engrafted with GMR-AML1-Luc/GFP cells were treated with vehicle control, MB and/or volasertib. Compared to vehicle control, although treatment with volasertib significantly reduced leukemia burden and spleen size, co-treatment with volasertib and MB was significantly even more effective in reducing the leukemia burden as well as in reducing the spleen size in NSG mice (Fig. 7C–E). Following 6 weeks of treatment, compared to vehicle control, treatment with volasertib or MB significantly improved survival of the NSG mice. Moreover, co-treatment with MB and volasertib was significantly more effective than volasertib alone in improving survival of NSG mice, again without inducing toxicity (Fig. 7F). These findings highlight the single agent in vivo efficacy of OM, MB and volasertib, as well as superior efficacy of co-treatment with MB and volasertib against the xenograft model of GMR-AML1 cells.

## Discussion

In this study, we present a comprehensive characterization of the first continuously cultured cell line (GMR-AML1) derived from BMA of a patient with FPD-MM. GMR-AML1 faithfully retains the RUNX1 mutation found in the original FPD-MM stage in the patient, making it a valuable tool for investigating disease mechanisms and therapeutic interventions. The biologic significance of the germline RUNX1 K194N mutation is highlighted by the observation that the other pedigree members with the same germline mutation had developed FPD or FPD-MM. The GMR-AML1 cell line exhibits a unique genetic co-mutation profile with mutations detected at the high VAF level distinct from the patient’s FPD-MM cells, suggestive of clonal expansion and adaptation during the in vitro culture. WES of GMR-AML1 cells revealed additional mutations in genes such as TP53, AIM2, NELFB, and others, indicating the role of these mutations in promoting in vitro survival and growth. GMR-AML1 cells expressed surface markers of AML stem cells [26, 29], providing insights into their differentiation status. GMR-AML cells induced an aggressive AML phenotype highlighted by splenic enlargement and lethal outcomes in approximately six weeks after engraftment in immune-depleted mice.

Consistent with a previous report on AML with mtRUNX1 [4], we demonstrate that depletion of RUNX1 using CRISPR KO in GMR-AML1 cells led to altered cell cycle distribution and reduced cell growth, supporting the critical role of RUNX1 in maintaining cellular homeostasis. In GMR-AML1 cells, treatment with HHT was lethal and induced synergistic lethality with venetoclax. The presence of active super-enhancers of MYB, MYC, CDK6 and BCL2, associated with increased gene-expressions of MCL1 and Bcl-xL may explain why GMR-AML1 cells were relatively resistant to monotherapy with venetoclax and with MCL1 or Bcl-xL-specific inhibitors [21–23]. Synergy of co-treatment with HHT and venetoclax may be explained by HHT-induced decline in active chromatin and mRNA expressions of genes involved in protein translation and of c-Myc targets, as well as in protein levels of MCL1, Bcl-xL and BFL1 in GMR-AML1 cells. These findings are consistent with previous reports showing lethal activity of HHT and venetoclax in AML with somatic mtRUNX1 [12]. Additionally, similar to HHT, the tubulin antagonist and cell-cycle G2/M phase-arresting agent MB, also reduced gene-expressions associated with cell cycle progression and protein translation, as well as induced in vitro loss of viability of GMR-AML1 cells. Based on our findings that MB treatment increases PLK1 levels in GMR-AML1 cells, and both MB and PLK1 inhibitor volasertib reduce nascent peptide elongation, co-treatment with MB and volasertib also exerted synergistic in vitro lethality in GMR-AML1 cells. Consistent with this, monotherapy with omacetaxine (HHT), MB, and volasertib demonstrated efficacy in the xenograft mouse model of GMR-AML1 cells by reducing leukemia burden and improving overall survival. Co-treatment with MB and volasertib also exhibited superior in vivo efficacy, suggesting a potential synergistic therapeutic strategy for FPD-MM. Taken together, the establishment of the GMR-AML1 cell line addresses a critical need for developing relevant additional in vitro and in vivo models to study FPD-MM and develop targeted therapies.

Our findings also provide valuable insights into the genetic landscape, biological features, and potential therapeutic strategies for the rare and clinically challenging disorder of FPD-MM. ATAC-Seq and RNA-Seq analyses provided valuable information on the chromatin accessibility and gene expression changes associated with disease progression from RUNX1-FPD to FPD-MM. These analyses identified key loci with altered chromatin accessibility and gene expression, such as MECOM, PIM1, RELB, CDKN1B, CDK6, MYB, and BCL2, highlighting potential drivers of disease evolution. The increased activity of super-enhancers further underscores the regulatory changes driving the transition to FPD-MM. Our study extended to the investigation of HHT, mebendazole (MB) and other anti-mitosis agents as potential therapeutic options. HHT and MB treatments showed selective toxicity against FPD-MM compared to RUNX1-FPD cells. Notably, HHT and MB also reduced pro-growth and pro-survival oncoproteins in FPD-MM cells expressing AML stem cell markers. In FPD-MM cells, MB treatment reduced chromatin accessibility and gene expressions associated with cell cycle progression and protein translation, further supporting its role as a potential therapeutic agent in FPD-MM. Taken together, findings presented highlight the potential of targeted therapeutic interventions in FPD-MM, leveraging vulnerabilities induced by the RUNX1 mutation and the co-mutations. These targeted therapies could also then be potentially tested as chemo-preventive strategies to retard progression of RUNX1-FPD to FPD-MM.

In conclusion, our study presents a comprehensive characterization of the first FPD-MM GMR-AML cell line, providing insights into its genetic profile, biological features, and response to therapeutic agents. These findings offer new perspectives on the pathogenesis of FPD-MM and highlight potential avenues for the development of precision therapies targeting this challenging disorder. Moving forward, deeper exploration of the identified genetic alterations and their functional consequences could uncover new avenues for targeted therapies in FPD-MM with the goal to revert it to RUNX1-FPD [30].

### Supplementary information

Supplemental Figure Legends

Sypplemental Figures

Supplemental Materials and Methods

## Data Availability

Bulk ATAC Seq, single-cell ATAC-Seq, ChIP-Seq, bulk RNA-Seq and single-cell RNA-Seq datasets have been deposited in GEO as a Super Series and assigned Accession ID GSE252746.

## References

[1] Sood R, Kamikubo Y, Liu P (2017). Role of RUNX1 in hematological malignancies. Blood.

[2] Mangan JK, Speck NA (2011). RUNX1 mutations in clonal myeloid disorders: from conventional cytogenetics to next generation sequencing, a story 40 years in the making. Crit Rev Oncog.

[3] Metzeler KH, Bloomfield CD (2017). Clinical relevance of RUNX1 and CBFB alterations in acute myeloid leukemia and other hematological disorders. Adv Exp Med Biol.

[4] Mill CP, Fiskus W, DiNardo CD, Qian Y, Raina K, Rajapakshe K (2019). RUNX1 targeted therapy for AML expressing somatic or germline mutation in RUNX1. Blood.

[5] Homan CC, Drazer MW, Yu K, Lawrence DM, Feng J, Arriola-Martinez LA (2023). Somatic mutational landscape of hereditary hematopoietic malignancies caused by germ line RUNX1, GATA2, and DDX41 variants. Blood Adv.

[6] Brown AL, Hahn CN, Scott HS (2020). Secondary leukemia in patients with germline transcription factor mutations (RUNX1, GATA2, CEBPA). Blood.

[7] Schnittger S, Dicker F, Kern W, Wendland N, Sundermann J, Alpermann T (2011). RUNX1 mutations are frequent in de novo AML with noncomplex karyotype and confer an unfavorable prognosis. Blood.

[8] Tang JL, Hou HA, Chen CY, Liu CY, Chou WC, Tseng MH (2009). AML1/RUNX1 mutations in 470 adult patients with de novo acute myeloid leukemia: prognostic implication and interaction with other gene alterations. Blood.

[9] Gonzales F, Barthélémy A, Peyrouze P, Fenwarth L, Preudhomme C, Duployez N (2021). Targeting RUNX1 in acute myeloid leukemia: preclinical innovations and therapeutic implications. Expert Opin Ther Targets.

[10] Toratani K, Watanabe M, Kanda J, Oka T, Hyuga M, Arai Y (2023). Unrelated hematopoietic stem cell transplantation for familial platelet disorder/acute myeloid leukemia with germline RUNX1 mutations. Int J Hematol.

[11] Tsirigotis P, Byrne M, Schmid C, Baron F, Ciceri F, Esteve J (2016). Relapse of AML after hematopoietic stem cell transplantation: methods of monitoring and preventive strategies. A review from the ALWP of the EBMT. Bone Marrow Transpl.

[12] Mill CP, Fiskus W, DiNardo CD, Birdwell C, Davis JA, Kadia TM (2022). Effective therapy for AML with RUNX1 mutation by cotreatment with inhibitors of protein translation and BCL2. Blood.

[13] Subramanian A, Narayan R, Corsello SM, Peck DD, Natoli TE, Lu X (2017). A next generation connectivity map: L1000 platform and the first 1,000,000 profiles. Cell.

[14] Li Y, Thomas D, Deutzmann A, Majeti R, Felsher DW, Dill DL (2019). Mebendazole for differentiation therapy of acute myeloid leukemia identified by a lineage maturation index. Sci Rep..

[15] Meco D, Attinà G, Mastrangelo S, Navarra P, Ruggiero A (2023). Emerging perspectives on the antiparasitic mebendazole as a repurposed drug for the treatment of brain cancers. Int J Mol Sci.

[16] He L, Shi L, Du Z, Huang H, Gong R, Ma L (2018). Mebendazole exhibits potent anti-leukemia activity on acute myeloid leukemia. Exp Cell Res.

[17] Tontsch-Grunt U, Rudolph D, Waizenegger I, Baum A, Gerlach D, Engelhardt H (2018). Synergistic activity of BET inhibitor BI 894999 with PLK inhibitor volasertib in AML in vitro and in vivo. Cancer Lett.

[18] De Souza C, Madden J, Koestler DC, Minn D, Montoya DJ, Minn K (2021). Effect of the p53 P72R polymorphism on mutant TP53 allele selection in human cancer. J Natl Cancer Inst.

[19] Fiskus W, Mill CP, Nabet B, Perera D, Birdwell C, Manshouri T (2021). Superior efficacy of co-targeting GFI1/KDM1A and BRD4 against AML and post-MPN secondary AML cells. Blood Cancer J.

[20] Birdwell C, Fiskus W, Kadia TM, DiNardo CD, Mill CP, Bhalla KN (2021). EVI1 dysregulation: impact on biology and therapy of myeloid malignancies. Blood Cancer J.

[21] Loven J, Hoke HA, Lin CY, Lau A, Orlando DA, Vakoc CR (2013). Selective inhibition of tumor oncogenes by disruption of super-enhancers. Cell.

[22] Merino D, Kelly GL, Lessene G, Wei AH, Roberts AW, Strasser A (2018). BH3-mimetic drugs: blazing the trail for new cancer medicines. Cancer Cell.

[23] Diepstraten ST, Anderson MA, Czabotar PE, Lessene G, Strasser A, Kelly GL (2022). The manipulation of apoptosis for cancer therapy using BH3-mimetic drugs. Nat Rev Cancer.

[24] Fiskus W, Sharma S, Qi J, Valenta JA, Schaub LJ, Shah B (2014). Highly active combination of BRD4 antagonist and histone deacetylase inhibitor against human acute myelogenous leukemia cells. Mol Cancer Ther.

[25] Fiskus W, Cai T, DiNardo CD, Kornblau SM, Borthakur G, Kadia TM (2019). Superior efficacy of cotreatment with BET protein inhibitor and BCL2 or MCL1 inhibitor against AML blast progenitor cells. Blood Cancer J.

[26] Fiskus W, Mill CP, Birdwell C, Davis JA, Das K, Boettcher S (2023). Targeting of epigenetic co-dependencies enhances anti-AML efficacy of Menin inhibitor in AML with MLL1-r or mutant NPM1. Blood Cancer J.

[27] Fiskus W, Boettcher S, Daver N, Mill CP, Sasaki K, Birdwell CE (2022). Effective menin inhibitor-based combinations against AML with MLL rearrangement or NPM1 mutation (NPM1c). Blood Cancer J.

[28] Nagelreiter F, Coats MT, Klanert G, Gludovacz E, Borth N, Grillari J (2018). OPP Labeling enables total protein synthesis quantification in CHO Production cell lines at the single-cell level. Biotechnol J.

[29] Thomas D, Majeti R (2017). Biology and relevance of human acute myeloid leukemia stem cells. Blood.

[30] Eriksson A, Engvall M, Mathot L, Österroos A, Rippin M, Cavelier L (2023). Somatic exonic deletions in RUNX1 constitutes a novel recurrent genomic abnormality in acute myeloid leukemia. Clin Cancer Res.

