# Supplementary material for: Efficacy of novel agents against cellular models of familial platelet disorder with myeloid malignancy (FPD-MM)
Source: Blood Cancer J. 2024 Feb 5;14(1):25. doi: 10.1038/s41408-024-00981-4 (PMC10844204; doi:10.1038/s41408-024-00981-4)
Supplement: Supplementary file 1 — Supplemental Figure Legends [file 41408_2024_981_MOESM1_ESM.docx]

**Supplemental Figure Legends**

**Supplemental Figure 1. Determination of karyotype and absence of MLL rearrangement or MYC amplification in GMR-AML1 cells. A.** Pedigree chart of the patient and progeny for Runx1 mutation and disease state. **B.** Karyotype analysis of GMR-AML1 cells. **C.** FISH analysis for MLL rearrangement and MYC amplification. **D.** Epigenetic mutations identified by whole exome sequencing in GMR-AML1 cells. **E.** The log2 fold-decrease (> 1.25-fold) in accessibility of genes in FPD-MM vs. FPD as identified by ATAC-Seq analysis. **F.** Three longitudinal samples of FPD or FPD-MM were harvested and RNA was extracted. RNA-Seq analyses were performed. The heatmap shows mRNAs up or down-regulated ≥ 1.25-fold and p value < 0.05 in the FPD-MM relative to the FPD samples. **G.** mRNA expression of leukemia relevant genes in FPD-MM relative to FPD assessed by qPCR.

**Supplemental Figure 2. Differential Sensitivity of GMR-AML1 cells to chemotherapeutic and targeted agents.** **A.** GMR-AML1/Cas9 cells were transduced with lentivirus expressing sgRNA control (tdTomato) or Runx1 guide RNAs (eGFP) for 72 hrs. Cells were sorted by FACS into tdTomato or GFP positive populations and cultured for an additional four days. Following this, immunoblot analyses were conducted on total cell lysates. The expression of GAPDH in the lysates served as the loading control. **B.** GMR-AML1 cells were treated with the indicated concentrations of panobinostat (HDACi), A-1155463 (BCL-x_L_i), AZD 5991 (MCL1i), daunorubicin, etoposide, and cytarabine (Ara-C) for 48 hrs. Then, the percentage non-viable cells was determined by TOPRO-3 iodide staining and flow cytometry. **C.** Oncoplot of mutations identified by NGS in PD, FPD and FPD-MM samples utilized in these studies. **D.** NGS-identified Runx1 mutation(s) in PD, FPD and FPD-MM samples.

**Supplemental Figure 3. Treatment with HHT concordantly depletes ATAC accessibility and mRNA expression of REACTOME RNAPII transcription gene set in GMR-AML1 cells.** GMR-AML1 cells were treated with 100 nM of HHT for eight hours and ATAC-Seq and RNA-Seq analyses were performed. Log2 fold-change of selected concordant ATAC-Seq and mRNA expression alterations compared to REACTOME RNAPII Transcription in HHT-treated GMR-AML1 cells.

**Supplemental Figure 4. Treatment with HHT depletes mRNA expressions of cell cycle and MYC HALLMARK and REACTOME pathways in PD FPD-MM samples. A.** Primary FPD-MM18 cells were treated with 100 nM of HHT for 16 hours and single cell RNA-Seq (scRNA-Seq) analysis was performed. SingleR analysis was utilized to define cell clusters. Relative cell population represented in each cluster from untreated and HHT treated FPD-MM18 cells. **B-C.** Primary FPD-MM18 cells were treated with 100 nM of HHT for 16 hours and bulk RNA-Seq analysis was performed. Gene set enrichment analysis (GSEA) in HHT treated cells compared with HALLMARK and REACTOME pathway datasets. **D.** Primary FPD-MM18 cells were treated with 100 nM of HHT for 16 hours and bulk RNA-Seq analysis was performed. The volcano plot shows significance and Log2 fold expression changes of selected mRNAs. **G-I.** Primary FPD-MM17 cells were treated with 100 nM of HHT for 16 hours and bulk RNA-Seq analysis was performed. Gene set enrichment analysis (GSEA) in HHT treated FPD-MM17 cells compared with HALLMARK and REACTOME pathway datasets. **J.** Primary FPD-MM17 cells were treated with 100 nM of HHT for 16 hours and bulk RNA-Seq analysis was performed. The volcano plot shows significance and log2 fold expression changes of selected mRNAs. **K.** Comparison of baseline mRNA expression signature in GMR-AML1 vs. FPD-MM17 or FPD-MM18. Similar equals p value greater than 0.05; Less or greater indicates differential expression in FPD-MM17 or FPD-MM18 at p < 0.05. **L.** Comparison of HHT-treated mRNA expression signature (1.25-fold up or down and a p < 0.05) in GMR-AML1 vs. FPD-MM17 vs. FPD-MM18.

**Supplemental Figure 5. Treatment with Mebendazole is relatively sparing of normal CD34+ progenitor cells.** Normal CD34+ HPCs were treated with the indicated concentrations of Mebendazole (MB) for 96 hrs. At the end of treatment, the percentage non-viable cells was determined by TO-PRO-3 iodide staining and flow cytometry.

**Supplemental Figure 6. Treatment with MB negatively enriches MYC targets and rRNA processing gene sets. A-B.** GMR-AML1 cells were treated with 1,000 nM of MB for 16 hours and bulk RNA-Seq analysis was performed. Gene set enrichment analysis (GSEA) in MB treated cells compared with HALLMARK and REACTOME pathway datasets. **C.** GMR-AML1 cells were treated with the indicated concentrations of MB and/or Plogosertib for 48 hrs. Then the percentage annexin V-positive apoptotic cells were determined by flow cytometry. Delta synergy scores (ZIP) were determined using SynergyFinder v3.0. **D.** Normal CD34+ HPCs were treated with the indicated concentrations of Volasertib for 96 hrs. At the end of treatment, the percentage non-viable cells was determined by TO-PRO-3 iodide staining and flow cytometry. **E.** Normal CD34+ HPCs were treated with the indicated concentrations of Mebendazole (MB) and/or Volasertib for 96 hrs. At the end of treatment, the percentage non-viable cells was determined by TO-PRO-3 iodide staining and flow cytometry.
