# Supplementary material for: Efficacy of novel agents against cellular models of familial platelet disorder with myeloid malignancy (FPD-MM)
Source: Blood Cancer J. 2024 Feb 5;14(1):25. doi: 10.1038/s41408-024-00981-4 (PMC10844204; doi:10.1038/s41408-024-00981-4)

Supplementary Figure 1

A.

Pedigree Chart of GMR-AML1 Patient

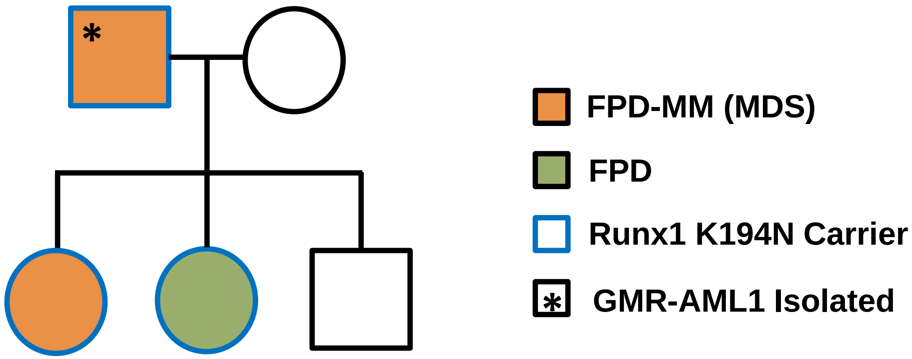

B.

Karyotype (Cell line - Normal 46,XY)

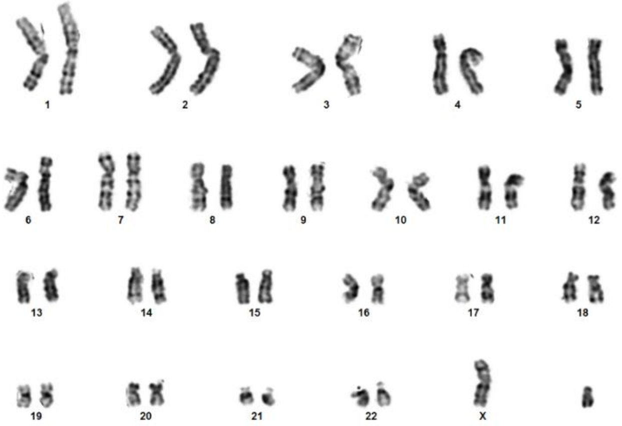

C.

FISH assay c-myc & MLL1/KMT2A (negative for arrangement)

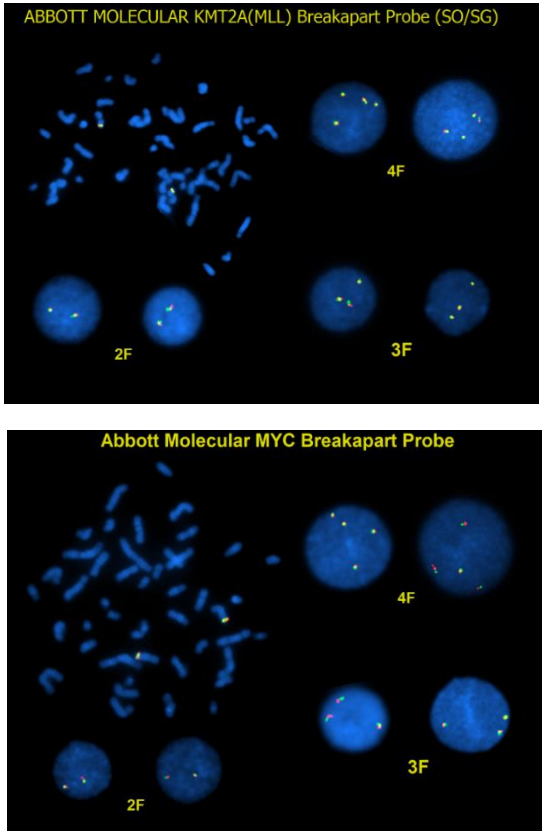

Supplementary Figure 1

Mutations/Variants of Unknown Significance (VUS) – Whole Exome Sequencing

D.

Gene	Mutation (cDNA)	Mutation (Protein)	Mutation Type	VAF (%)
**TP53**	c.C215G	p.P72R	nonsynonymous SNV	55
**AIM2**	c.1018delA	p.K340fs	frameshift deletion	52
**NELFB**	c.G1569C	p.L523F	nonsynonymous SNV	50
**CEP152**	c.C1110G	p.Y370X	stopgain SNV	49
**SUGP2**	c.A68T	p.H23L	nonsynonymous SNV	45
**RRM2B**	c.211dupC	p.R71fs	frameshift insertion	43
**TADA3**	c.C80G	p.T27R	nonsynonymous SNV	42
**SPDYE6**	c.G874T	p.G292C	nonsynonymous SNV	36
**PRDM9**	c.C2442A	p.S814R	nonsynonymous SNV	33

E.

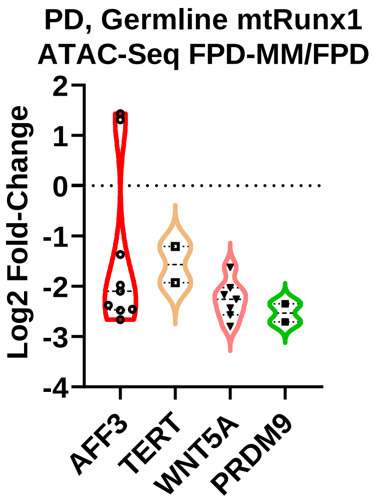

F.

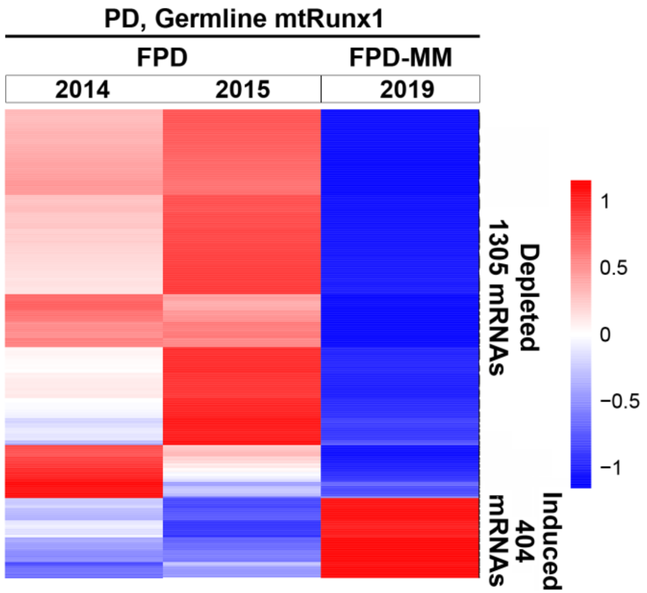

G.

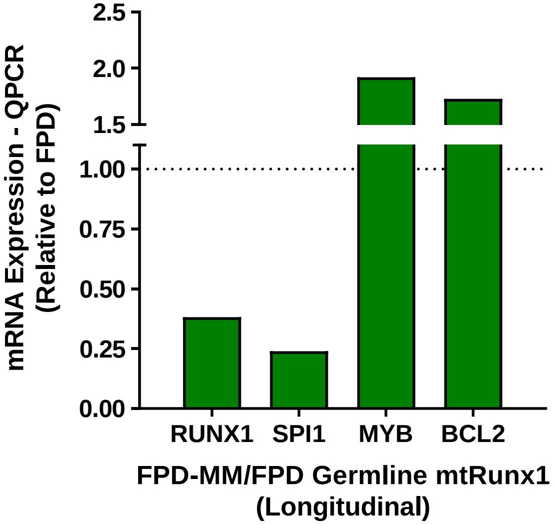

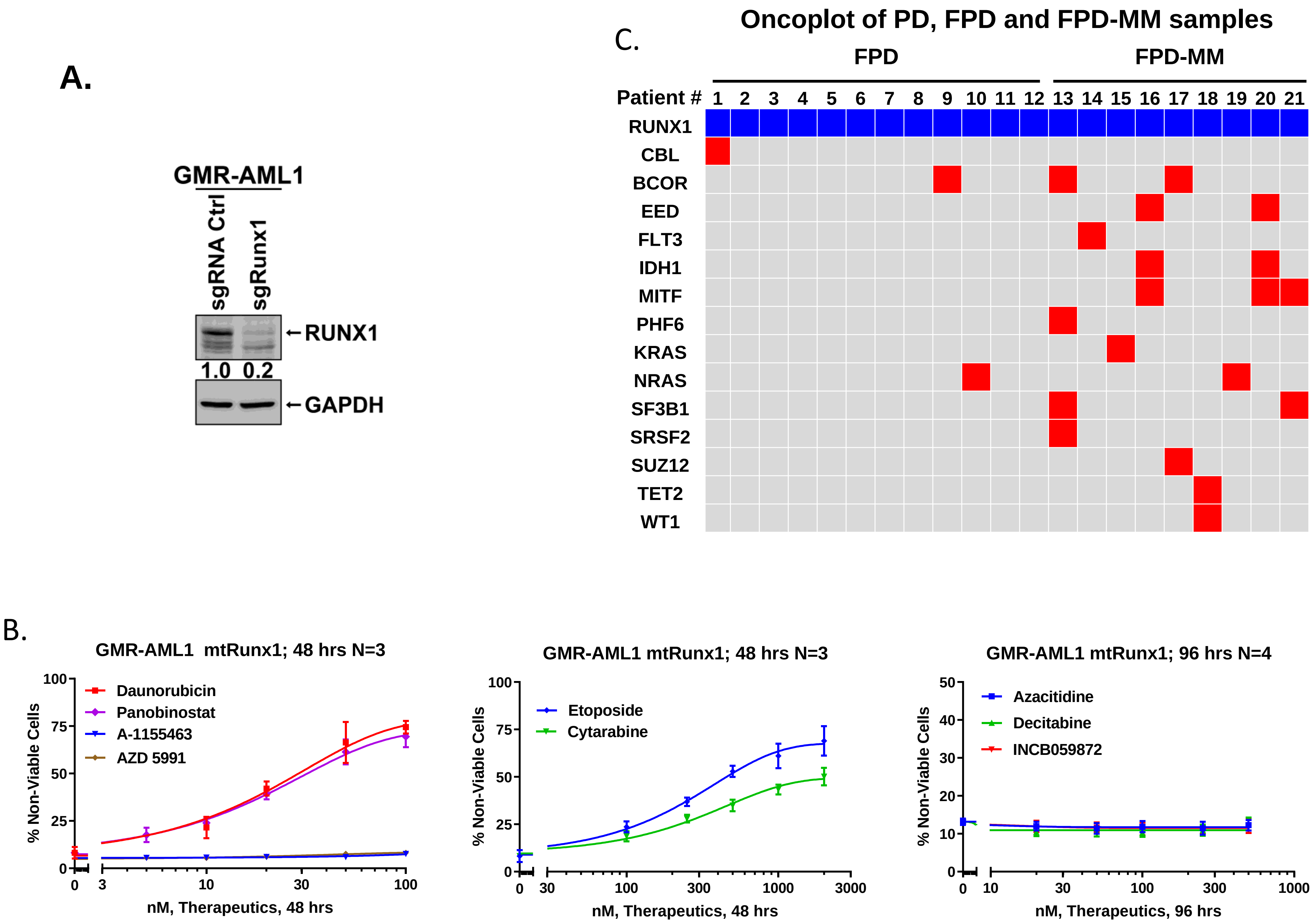

Supplementary Figure 2

Supplementary Figure 2

D.

Sample #	RUNX1 Mutation
**FPD-1**	Splice c.352-1 G>C
**FPD-2**	W279*
**FPD-3**	R166Q
**FPD-4**	K194N
**FPD-5**	Y189*
**FPD-6**	W279*
**FPD-7**	Deletion Ex1-5
**FPD-8**	Q335*
**FPD-9**	Splice c.352-1 G>T
**FPD-10**	R201*
**FPD-11**	p.Val164CysfsX49
**FPD-12**	W279*
**FPD-MM-13**	K194N
**FPD-MM-14**	L56S
**FPD-MM-15**	Deletion ≥ first six exons
**FPD-MM-16**	p.Val164CysfsX49
**FPD-MM-17**	R166*
**FPD-MM-18**	Q335*
**FPD-MM-19**	R201*
**FPD-MM-20**	p.Val164CysfsX49
**FPD-MM-21**	LOH; 2nd allele dominant clone S141L

Supplementary Figure 3

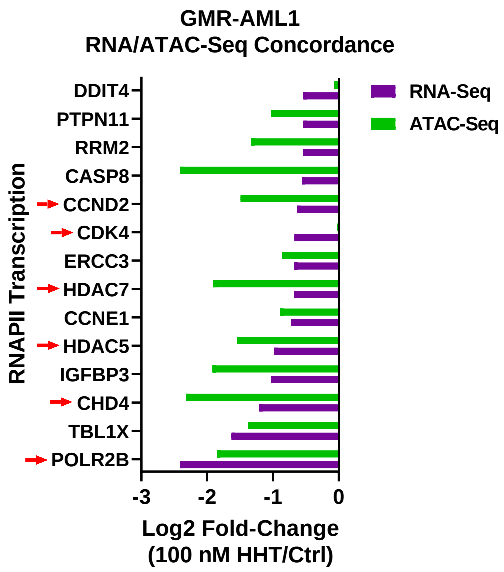

Supplementary Figure 4

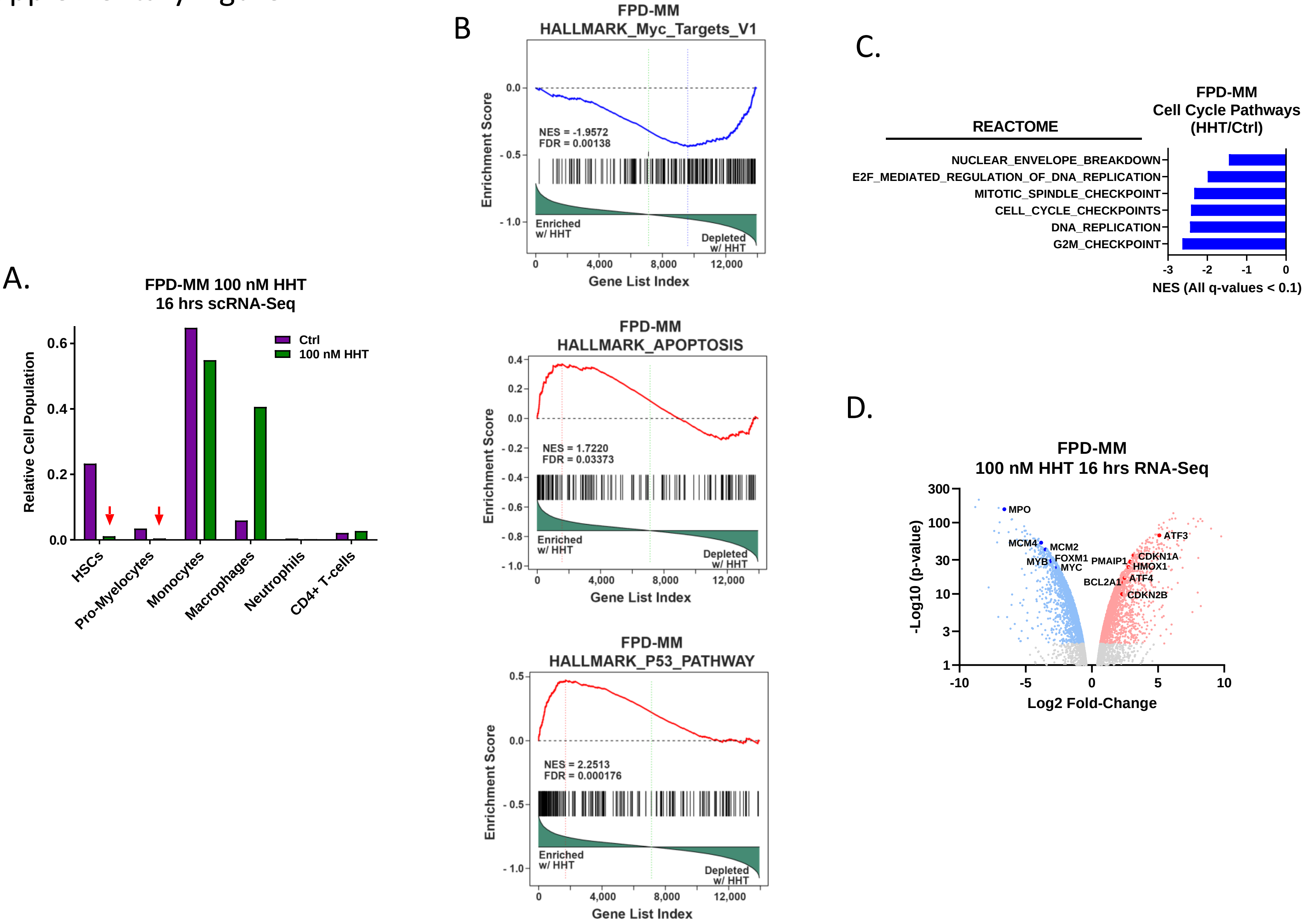

Supplementary Figure 4

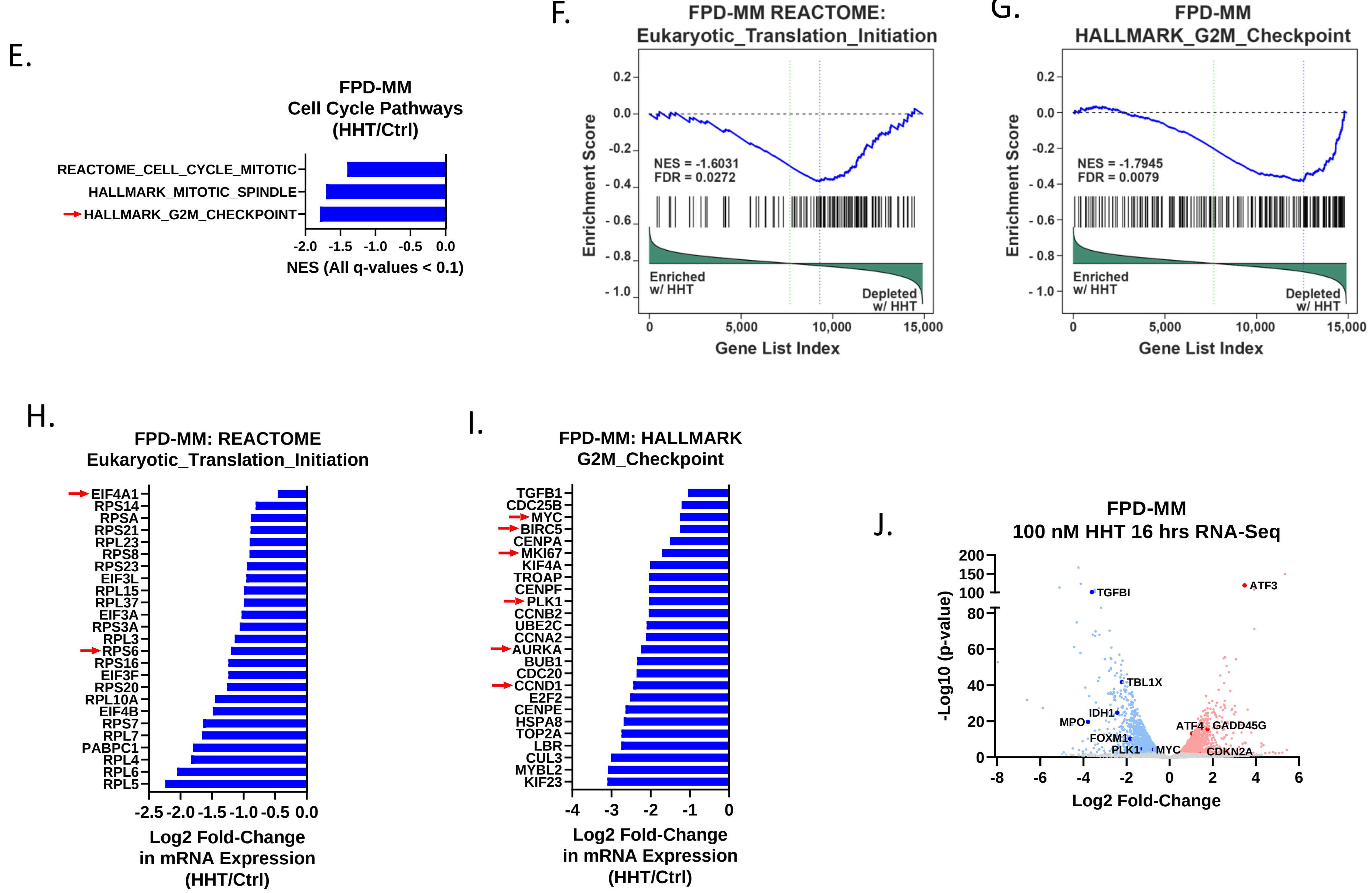

Supplementary Figure 4

K.

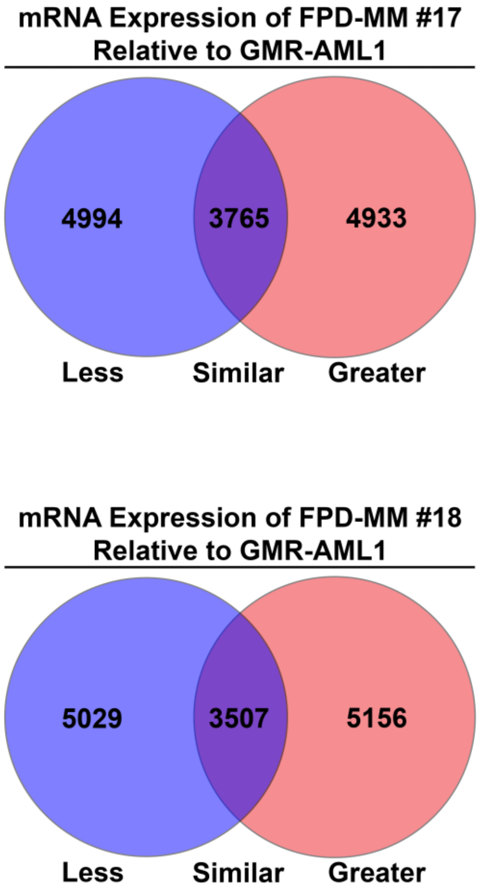

L.

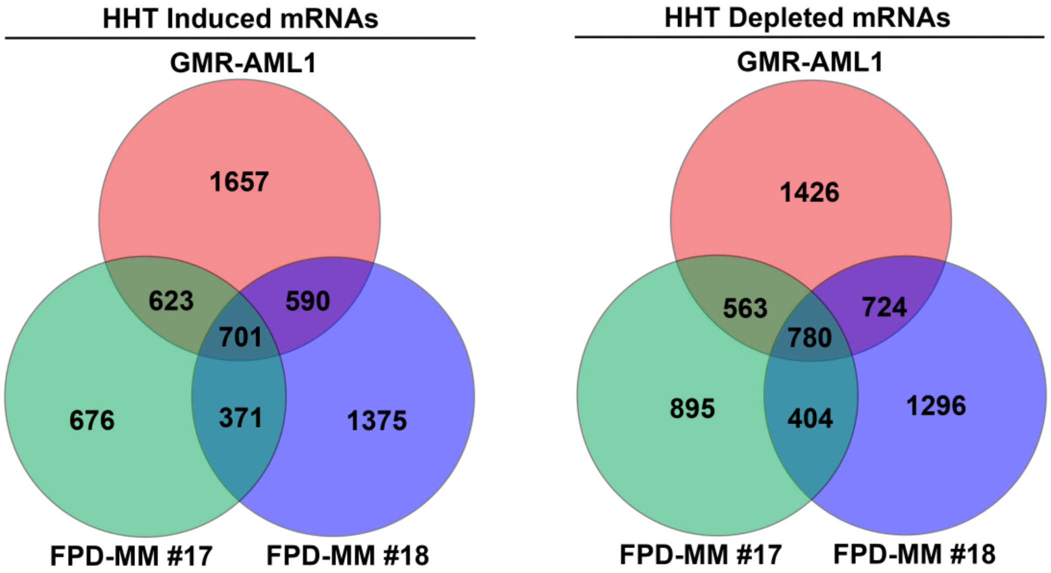

Supplementary Figure 5

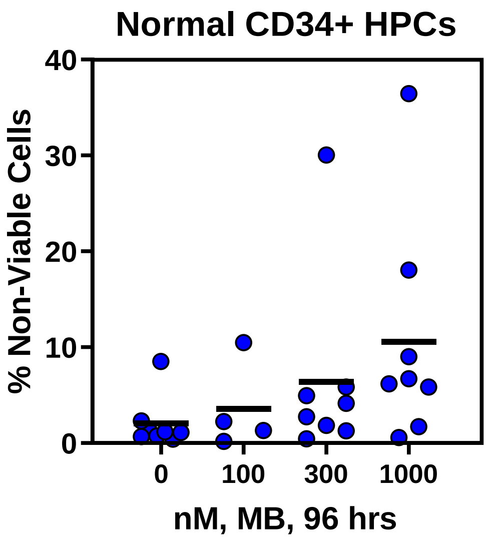

Supplementary Figure 6

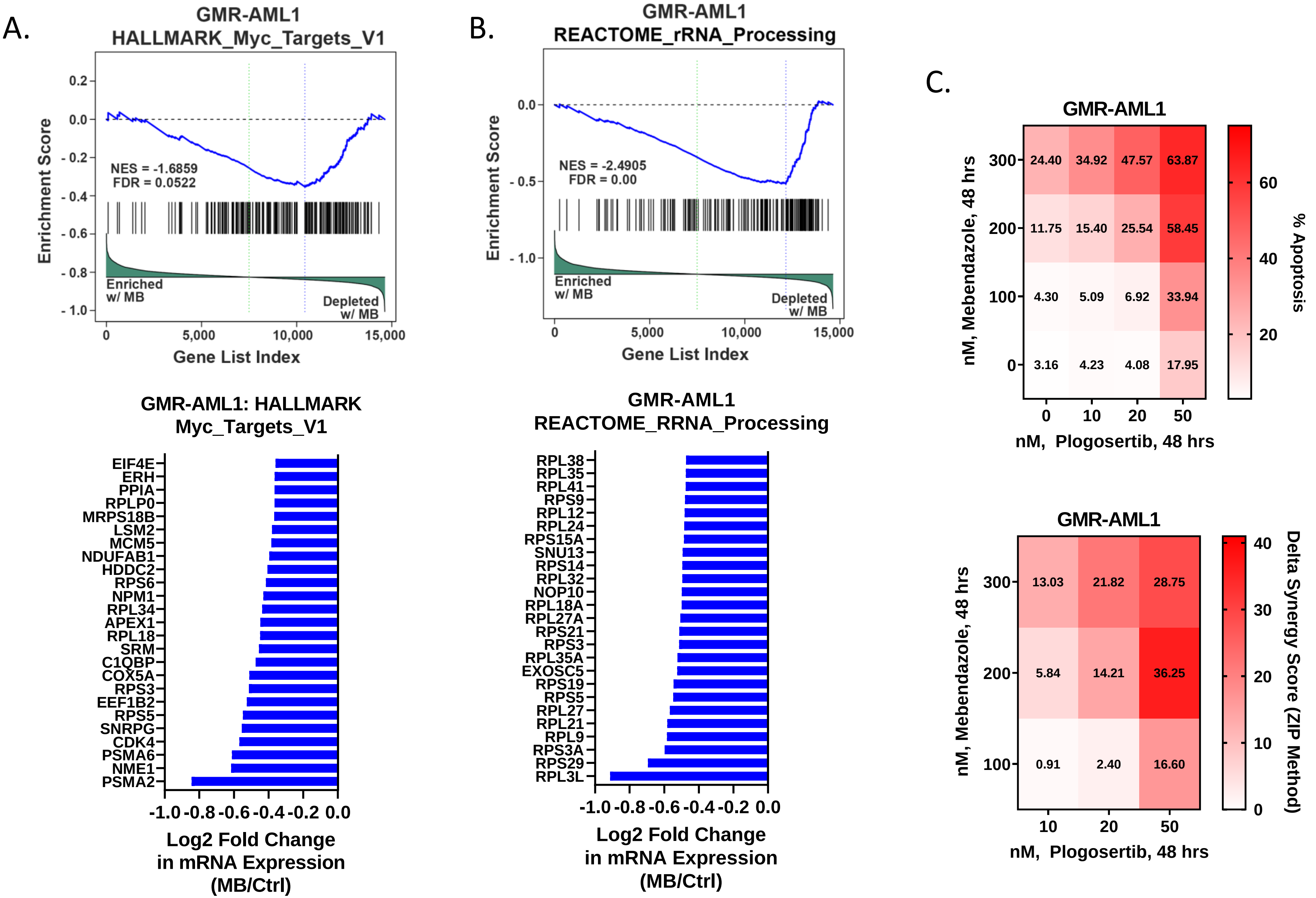

Supplementary Figure 6

D.

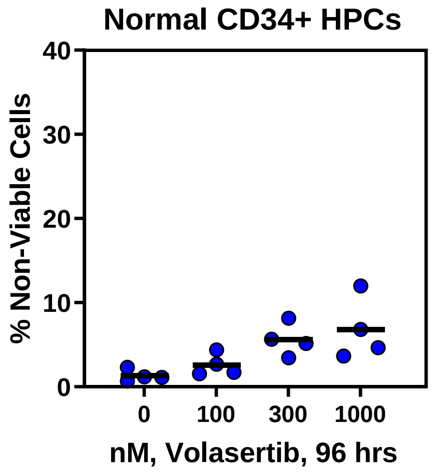

E.

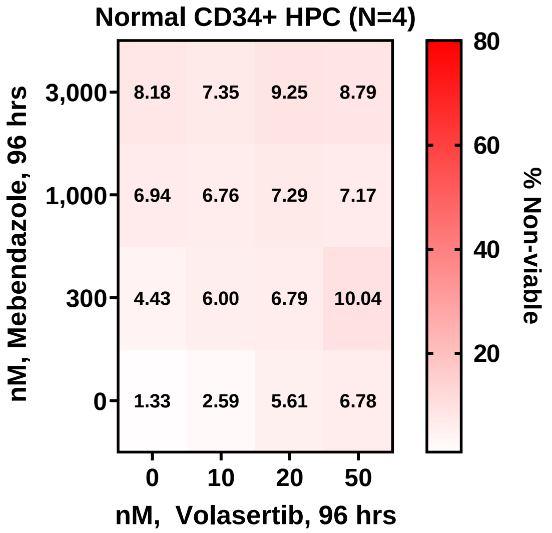

Supplement: Supplementary file 2 — Sypplemental Figures [file 41408_2024_981_MOESM2_ESM.pdf]
