# Supplementary material for: Efficacy of novel agents against cellular models of familial platelet disorder with myeloid malignancy (FPD-MM)
Source: Blood Cancer J. 2024 Feb 5;14(1):25. doi: 10.1038/s41408-024-00981-4 (PMC10844204; doi:10.1038/s41408-024-00981-4)
Supplement: Supplementary file 3 — Supplemental Materials and Methods [file 41408_2024_981_MOESM3_ESM.docx]

**Supplemental Methods:**

**Contact for Reagent sharing. Kapil N Bhalla. Department of Leukemia, MD. Anderson Cancer Center, 1400 Holcombe Blvd, Unit428, Houston, TX, 77030. kbhalla@mdanderson.org**

**Reagents and antibodies.** Homoharringtonine, Mebendazole, Volasertib, Plogosertib, Venetoclax, Fenbendazole, Flubendazole, Daunorubicin, Panobinostat, A-1155463, AZD 5991, Cyarabine, Azacitidine, Decitabine, INCB059872, and Alisertib were obtained from MedChem Express (Monmouth Junction, NJ). Etoposide was obtained from Selleck Chemicals LLC (Houston, TX). All compounds were prepared as 10 mM stocks in 100% DMSO and frozen at -80°C in 5-10 µL aliquots to allow for single use, thus avoiding multiple freeze-thaw cycles that could result in compound decomposition and loss of activity. anti-Bak [RRID: AB_10828597], anti-Bax [RRID: AB_10557411], anti-BFL-1/A1 [RRID: AB_2798390], anti-BIM [RRID: AB_1030947], anti-CBFβ [RRID: AB_2722525], anti-CDK4 [RRID: AB_2631166], anti-EVI1 [RRID: AB_284098], anti-c-Myb [RRID: AB_2716637], anti-c-Myc [RRID: AB_1903938], anti-MCL-1 [RRID: AB_2799149], anti-Notch1 [RRID: AB_2153354], anti-Plk1 [RRID: AB_2167409], anti-PU.1 [RRID: AB_10693421], anti-RUNX1/(AML1) [RRID: AB_10859035], anti-RUNX3 [RRID: AB_2798118], antibodies were obtained from Cell Signaling Technologies (Beverly, MA). Anti-BCL2 [RRID: AB_626733], anti-Bcl-xL [RRID:AB_626739], anti-CDK6 [RRID: AB_10610066], anti-GAPDH [RRID: AB_627679], and anti-β-Actin [RRID: AB_626630] antibodies were obtained from Santa Cruz Biotechnologies (Dallas, TX). anti-SF3BI [RRID: AB_2880946] antibody was obtained from Proteintech (Rosemont, IL).

**Primary FPD and FPD-MM and normal CD34+ progenitor samples :** Patient-derived FPD and FPD-MM samples (peripheral blood and bone marrow aspirate) for the conduct of preclinical studies and for the creation of a cell line were obtained with informed consent under a clinical protocol approved by the MD Anderson Cancer Center’s Institutional Review Board (IRB# PA14-0392). Normal hematopoietic progenitor cells (HPCs) were obtained from delinked, de-identified cord blood samples. Mononuclear cells were purified by Ficoll Hypaque (Axis Shield, Oslo, Norway) density centrifugation following the manufacturer’s protocol. Mononuclear cells were washed once with sterile 1X PBS and suspended in complete RPMI media containing 20% FBS and counted to determine the number of cells isolated prior to immuno-magnetic selection. CD34+ AML blast progenitor cells were purified by immuno-magnetic beads conjugated with anti-CD34 antibody following the manufacturer’s protocol (StemCell Technologies, Vancouver, British Columbia) prior to utilization in the cell viability assays, RNA expression, and immunoblot analyses.

**Generation of a germline mutant Runx1 cell line (GMR-AML1).** The cell suspension of the original patient cells was adjusted to a concentration of 2 × 10^6^/mL in RPMI-1640 media with 20% FBS, 1% Pen-Strep, and 1% non-essential amino acids. Cells were incubated in a humidified incubator at 37°C and 5% CO_2_ in air. Media was changed once per week until cells began to proliferate and grow in small floating clusters. When cells began to proliferate (less than two months in culture) and change the color of the media more frequently, media was changed twice per week by dilution or by centrifugation at 200 x g for 5 minutes. Early passages of the cells were cryopreserved in 90% FBS + 10% DMSO in 5-10 million cell aliquots and stored in liquid nitrogen to allow for characterization of the cell line and to monitor for genetic drift. The presence of germline Runx1 mutation was confirmed via Sanger sequencing*^1^*.

**Cell lines and cell culture.** OCI-AML5 [DSMZ Cat# ACC-247, RRID:CVCL_1620] and OCI-AML2 [DSMZ Cat# ACC-99, RRID:CVCL_1619] cells were obtained from the DSMZ. HEK-293T cells were obtained from the Characterized Cell Line Core Facility at M.D. Anderson Cancer Center, Houston TX. All experiments with cell lines were performed within 6 months after thawing or obtaining from DSMZ. The cell lines were also authenticated in the Characterized Cell Line Core Facility at M.D. Anderson Cancer Center, Houston TX. OCI-AML2 and OCI-AML5 were cultured in ribonucleoside-containing Alpha-MEM media with 20% FBS, 1% non-essential amino acids (NEAA), and 1% penicillin/streptomycin. OCI-AML5 cells were supplemented with 10 µg/mL GM-CSF. HEK-293T cells were cultured in high-glucose-formulated DMEM media with 10% FBS, 1% NEAA, 1% L-glutamine, and 1% penicillin/streptomycin. Logarithmically growing, mycoplasma-negative cells were utilized for all experiments. Following drug treatments, cells were washed free of the drug(s) prior to the performance of the studies described.

**Cell Line Authentication**. The cell lines utilized in these studies were authenticated in the Characterized Cell Line Core Facility at M.D. Anderson Cancer Center, Houston TX utilizing STR profiling.

**Sequencing of primary de novo FPD and FPD-MM cells:** We performed targeted next-generation sequencing (NGS) of DNA samples from bone marrow or peripheral blood collected from patients at our center with FPD or FPD-MM *^2^*. Diagnostic bone marrow samples were obtained for mutational analysis. Total genomic DNA was extracted from unenriched peripheral blood (PB) or bone marrow (BM) samples using ReliaPrep genomic DNA isolation kit (Promega Corp, Madison, WI, USA). Briefly, a total of 250 ng of DNA was utilized to prepare sequencing libraries using Agilent HaloPlex custom Kit (Agilent Technologies, Santa Clara, CA, USA). The entire coding sequences of 81 genes including ABL1, ASXL1, BRAF, CALR, DNMT3A, EGFR, EZH2, FLT3, GATA1, GATA2, HRAS, IDH1, IDH2, KIT, KRAS, MDM2, IKZF2, JAK1, JAK2, MLL, MPL, MYD88, NOTCH1, NF1, NPM1, NRAS, PTPN11, RUNX1, TET2, TP53, and WT1 were interrogated on a custom-designed next-generation sequencing approach using the Illumina MiSeq platform (Illumina; San Diego, CA, USA; RRID:SCR_016379). The genomic reference sequence used was genome GRch37/hg19. The following software tools were utilized in the experimental setup and data analysis: Illumina Experiment Manager 1.6.0 (Illumina; San Diego, CA, USA), MiSeq Control Software 2.4 (Illumina; San Diego, CA, USA), Real Time Analysis 1.18.54 (Illumina; San Diego, CA, USA), Sequence Analysis Viewer 1.8.37 (Illumina; San Diego, CA, USA), MiSeq Reporter 2.5.1 (Illumina; San Diego, CA, USA), and SureCall 3.0.1.4 (Agilent Technologies; Santa Clara, CA, USA). A minimum of 80% reads at quality scores of AQ30 or higher were required to pass quality control. The lower limit of detection of this assay (analytical sensitivity) for single nucleotide variations was determined to be 5% (one mutant allele in the background of nineteen wild type alleles) to 10% (one mutant allele in the background of nine wild type alleles). Testing of patients with active hematologic malignancies was limited to somatic mutations only. The germline nature of RUNX1 mutations in FPD and FPD-MM samples was confirmed by targeted sequencing of a skin punch biopsy.

**Analysis of epigenetic state in AML cells *in vitro***. We determined the H3K27Ac [RRID:AB_2793305] status in GMR-AML1 by ChIPmentation following a previously described protocol*^3^*, with modifications on the concentration of AmpPure XP beads utilized for dual AmpPure XP SPRI bead selection of the final libraries. We utilized 0.65X beads for the first selection, then a 1.0X bead concentration to narrow the fragment size of the final tagmented ChIP DNA library. ChIP input DNA libraries were only selected with a 1.0X bead concentration. The individual libraries (ChIP and input) were quantified and quality-checked with Qubit and Bioanalyzer 2100 analysis, respectively. The libraries were pooled into one tube, purified utilizing a Qiagen MinElute column, and eluted in 20 µL for loading onto a NextSeq500 sequencer utilizing a mid-output kit. Raw sequence data were mapped to UCSC hg38 (NCBI 51) and log2 fold-changes were calculated with diffReps*^4^* [diffReps, RRID:SCR_010873]. Sequence tracks were visualized with IGV software*^5, 6^* [RRID:SCR_011793]. To identify super enhancers, we performed a ranked order of super enhancers (ROSE) analysis [ROSE, RRID:SCR_017390] utilizing the H3K27Ac status of the chromatin according to the methods of Loven et al.*^7^*. Analysis of transcription factor binding motifs in gained ATAC-Seq peaks was performed with HOMER [HOMER, RRID:SCR_010881].

**Transcriptome Analysis.** Total RNA was isolated from primary FPD, FPD-MM or GMR-AML1 cells treated with 100 nM homoharringtonine for 8 hrs (16 hrs primary) or 1,000 nM mebendazole for 16 hrs (24 hrs primary) utilizing a PureLink RNA Mini kit from Ambion, Inc. (Austin, TX). Sequencing libraries were prepared with ERCC spike-in controls in the MD Anderson Cancer Center DNA Sequencing and Microarray core facility and sequenced on an Illumina HiSeq-4000 next generation sequencer [Illumina HiSeq 3000/HiSeq 4000 System, RRID:SCR_016386]. Each library yielded 30-40 million read pairs. Data was mapped using STAR and Samtools (STAR [RRID:SCR_004463], SAMTOOLS [RRID:SCR_002105])*^8, 9^* onto the human genome build UCSC hg38 (NCBI 51) for human data. Gene expression was assessed using DESeq2*^10^* [DESeq2, RRID:SCR_015687], then variance stabilization and quantile normalization were applied. We considered that significance was achieved for fold changes greater than or equal to 1.25X up or down relative to the untreated or parental cells, and p-values less than 0.05. The final p-values were adjusted using the Benjamini & Hochberg method*^11^*. We inferred enriched pathways using the Gene Set Enrichment (GSEA) method*^12^*, and the gene set collection from the Molecular Signature Database (MSigDB)*^13^* [Molecular Signatures Database, RRID:SCR_016863]. Concordance between ATAC and RNA-Seq data and Venn diagrams were determined using standard bioinformatic pipelines*^14^*. Circos plots were generated following the methods of Krzywinski et al. 2009*^15^*.

**Whole Exome Analysis of GMR-AML1.** Whole exome analysis was performed on GMR-AML1 cells utilizing Agilent Exome 7 (SureSelect Human All Exon v7). The raw paired-end (PE) reads in FASTQ format was aligned to the human reference genome (hg38) for human DNA-Seq, using BWA alignment software. GMR-AML1 mutant calls were cataloged and are reported.

**scRNA-Seq Analysis of PD, FPD-MM Cells.** Primary FPD-MM cells were treated with 100 nM HHT for 16 hrs or 1,000 nM mebendazole for 24 hrs and then single cell separated over a Chromium controller (10X Genomics; Pleasanton, CA). Pre-amplification, indexing, and library preparations were conducted following the manufactures recommendation (10X Genomics). Final libraries were sequenced on an llumina NovaSeq 6000 Sequencing System (RRID:SCR_020150) (Illumina, San Diego, CA). Raw scRNA-seq data was pre-processed, de-multiplexed, and aligned to human reference genome (GRCh38) using CellRanger (10X Genomics). Cells with few (<200) or many (>6,000) genes, likely doublets or multiplets predicted by Scrublet (Wolock et al. 2019), and cells with >20% of read counts derived from the mitochondrial genome were removed. The batch effects were corrected by Harmony*^16^* and Seurat v4*^17^*. Raw unique molecular identifier (UMI) counts were log-normalized and used for principal component analysis (PCA). Seurat v4*^17^* was applied to the normalized gene-cell matrix to identify highly variable genes for unsupervised cell clustering. For visualization, the dimensionality was further reduced using Uniform Manifold Approximation and Projection (UMAP)*^18^* method. We also sub-clustered cells within each lineage to identify transcriptomically distinct subpopulations. To define the major cell type and state of each single cell, the top 50 most significant differentially expressed genes (DEGs) were identified for each cell cluster. We also identified DEGs for cell subpopulations of interest using Seurat which was then filtered to select significant DEGs (log2 fold change >1.0 or <-1.0 and FDR p-value <0.05) between two conditions. For pathway analysis, the curated gene sets (including Hallmark, GO, KEGG, REACTOME gene sets) was downloaded from the Molecular Signature Database, and single-sample GSVA was applied and pathway scores was calculated for each cell type using the GSVA software package *^19^*. Gene set enrichment analysis using GSEA software package*^12^* was performed to identify significantly enriched signaling pathways (FDR p-value < 0.05) between two conditions.

**Integrated analysis of scRNA-seq and scATAC-seq data.** For paired scATAC-seq data, Signac (v1.9.0)*^20^* was used to process the fragment files. We removed low-quality cells based on peak region fragments, nucleosome signal and TSS enrichment. The accessible chromatin peaks were called by using MACS2*^21^* method with the default parameters of Signac’s CallPeaks function. After removing peaks in non-standard chromosomes and in genomic blacklist regions, we quantified counts in each peak using the FeatureMatrix function. Then, we performed latent semantic indexing (LSI) and normalized with term-frequency inverse-document-frequency (TFIDF). RunSVD function was used on the TFIDF matrix to consequent dimensionality reduction with singular value decomposition (SVD). scATAC-seq and scRNA-seq data were integrated by using Seurat. For each cell type, we found differentially accessible peaks between groups by the FindAllMarkers function. And based on the filtered differentially accessible chromatin regions (log2 fold change >1.0 or <-1.0 and FDR p-value <0.05), we performed enriched motifs analysis in these genomic regions by HOMER*^22^*. We used filterbarcodes command of sinto (v0.9.0, https://timoast.github.io/sinto/) to get bam files for interested cell type, and generated bigWig files by using bamCoverage command of deeptools*^23^*. BigWig files were uploaded to the UCSC genome browser*^24^* for visualization.

**Plasmid Generation, Viral Packaging, and Creation of Cell Lines.** Plasmid constructs for the production of lentivirus were transfected with packaging plasmids psPAX2 and pMD2.G into HEK-293T cells utilizing jetPRIME reagent (PolyPlus Transfection, New York, NY). The psPAX2 and pMD2.G packaging plasmids were a gift from Didier Trono (Addgene plasmid #12260 and #12259 [RRID: Addgene_12260; RRID: Addgene_12259]). Media was changed the following day. Viral supernatant was collected 72 hours post transfection and filtered through a 0.45 µm PES membrane. AML cells were seeded at 5 x 10^5^ cells/mL in a 50:50 mix of media and lentiviral supernatant with 8 µg/mL polybrene (Sigma-Aldrich). The following day, the viral supernatant was removed by centrifugation and cells were transduced with fresh viral supernatant for an additional 24 hours. lenti-SpCas9 neo was a gift from Brett Stringer (Addgene #104996; RRID:Addgene_104996). Cas9-Neo lentivirus was produced as described above and transduced into GMR-AML1 cells. GMR-AML1 cells were then selected with 400 µg/mL G418. LRG (Lenti_sgRNA_EFS_GFP) was a gift from Christopher Vakoc (Addgene #65656; RRID:Addgene_65656). The Runx1 sgRNA oligonucleotides were designed utilizing the CHOP-CHOP prediction algorithm*^25^* and were purchased from Sigma. Guide RNAs were developed against exon 4 and exon 5 sequence of Runx1b. The ssDNA oligonucleotides were annealed (10 mM Tris pH 8.0, 50 mM NaCl, 1 mM EDTA pH 8.0) in a thermocycler by incrementally decreasing the temperature from 95 to 10 ^°^C (0.6 ^o^C every 150 sec). Runx1 sgRNA dsDNA was then ligated into Esp3I digested LRG. Runx1 sgRNA lentivirus was produced as described above and transduced into GMR-AML1/Cas9 expressing cells. GMR-AML1/Cas9 cells were then sorted by flow cytometry (FACSAria, FL-1 channel) for GFP positive and negative cells. To generate luciferase-expressing GMR-AML1 pHIV-Luc-ZsGreen (a gift from Bryan Welm [Addgene plasmid #39196; RRID: Addgene_39196]) was packaged as above and transduced into GMR-AML1 cells. GMR-AML1 cells were expanded and sorted by flow cytometry (FACSAria, FL-1 channel, top GFP-expressing cells) for their utilization in therapeutic in vivo mouse studies.

**CRISPR/Cas9-mediated Runx1 Knockout and in vitro or in vivo Competition Assay in GMR-AML1 cells.** To study the effects of knockout/depletion of Runx1 in GMR-AML1 cells, a stable Runx1 guide expressing system was developed as described above. For sgRNA control, eGFP was replaced with tdTomato via standard subcloning methods. tdTomato was derived from pHIV-dTomato, a gift from Bryan Welm (Addgene RRID:Addgene_21374). Gene-edited (GFP+) and non-edited (GFP-; Ctrl) GMR-AML1 cells were sorted three days post transduction and then combined at a 50:50 ratio and cultured for 30 days. The cells were passaged every 3-4 days. Knockout of Runx1 was confirmed by Western blot analysis 15 days post-transduction (after sorting). For in vivo, Runx1 KO (GFP+) and non-edited (tdTomato; sgRNA Ctrl) GMR-AML1 cells were sorted three days post transduction and then cultured for four additional days. Knockout of Runx1 was confirmed by Western blot analysis seven days post-transduction (after sorting). Female NOD.Cg-Prkdc^scid^ Il2rg^tm1Wjl^/SzJ (NSG) mice (stock number: 005557; 4-6 weeks of age) [Jackson Labs, Bar Harbor, ME; RRID: IMSR_JAX:005557] were exposed to 2.5 Gy of radiation. The following day, GMR-AML1 cells that had been transduced with either sgRNA Ctrl or sgRunx1 Ex5 were injected individually or at a 50:50 ratio in the lateral tail vein (1 million cells) of NSG mice. Engraftment of GMR-AML1 in the 50:50 experiment was determined at two weeks (2 mice) by flow cytometry for tdTomato or GFP and the remaining mice (N = 5 of each cohort) were euthanized once moribund. All in vivo studies were approved by and conducted in accordance with the guidelines of the IACUC at the M.D. Anderson Cancer Center, an AAALAC-accredited facility.

**Confocal immunofluorescent microscopy.** GMR-AML1 treated with 1,000 nM mebendazole for 16 hrs were cytospun onto glass slides to prepare for confocal microscopy. Cells were fixed with 4% paraformaldehyde in 1X PBS for 10 minutes, then washed three times with 1X PBS. Next, cells were permeabilized with 0.5% Triton X-100 for 5 minutes, then rinsed three times with 1X PBS. Cells were blocked in 3% BSA/PBS + 1% FBS for 1 hour at room temperature. Next, the primary antibody AlexaFluor 488 conjugated to β-Tubulin (D-10) [sc-5274; RRID:AB_2288090] was added and incubated in a humidified chamber overnight at 4° C. Excess antibody was removed by washing three times with 1X PBS. Nuclei were counterstained with DAPI (#62248, ThermoFisher), and then coverslips were mounted onto the slides utilizing Prolong Diamond anti-fade mountant (P36970, LifeTech, Carlsbad, CA). Imaging was performed on an Andor Revolution XDi WD spinning disk confocal microscope in the MD Anderson Flow Cytometry and Imaging Core. Images were obtained with a 60X oil immersion objective. Confocal analysis was performed at least twice. Representative images are shown for each condition.

**Cell cycle analysis of AML cells**. GMR-AML1 cells transduced with sgRNA Runx1, sorted (GPF negative and positive), and incubated for a total of 15 days or treated with mebendazole for 24 hours were harvested by centrifuging at 125 x g for 5 minutes. Cells were washed twice with 1× phosphate-buffered saline (PBS) in 12 x 75 flow tubes, re-suspended in 200 µL of 1X PBS and fixed in 70% ethanol by adding 800 µL of molecular grade 70% ethanol dropwise to the cells in the tube. The tubes were then vortexed to mix and stored overnight at -20°C. Fixed cells were washed twice with 1× PBS by centrifuging at 125 x g for 5 minutes and then stained in 250 µL of DNA staining buffer [5 mL Triton-PBS (100 µL of Triton X100 in 100 mL of 1X PBS) with 100 µL of 1 mg/mL propidium iodide and 100 µL of 10mg/mL RNAse A] in the dark for 15 minutes at 37°C. Cell-cycle data were collected on a flow cytometer with a 488 nM laser in the FL-2 channel and analyzed with Accuri CFlow6 software (BD Biosciences).

**Flow cytometry analysis of cell surface markers on GMR-AML1 cells.** To determine the immuno-phenotype of the GMR-AML1, cells were washed with 1X PBS and centrifuged at 200 x g for 5 minutes. The cells were suspended in 100 µL of 0.5% BSA/PBS and stained with fluorophore-conjugated anti-CLL1 [RRID: AB_11153496], anti-CD99 [RRID: AB_396039], anti-CD34 [RRID: AB_396151], anti-CD38 [RRID: AB_395853], anti-CD117 [RRID: AB_398461], anti-CD135 [RRID: AB_2738240], anti-CD123 [RRID: AB_2738305], anti-CD33 [RRID: AB_10896330], anti-CD244 [RRID: AB_11153502], anti-TIM3 [RRID: AB_2716866], anti-MPO [RRID: AB_2146481], anti-CD14 [RRID: AB_395797], anti-CD11b [RRID: AB_394774], anti-CD86 [RRID: AB_396012], anti-CD5 [RRID: AB_398594], anti-CD19 [RRID: AB_395812], anti-CD20 [RRID: AB_314258], anti-CD4 [RRID: AB_395751], anti-CD3 [RRID: AB_2739765] or IgG-isotype controls (BD Biosciences, San Jose, CA) for 30 minutes on ice. Cells were washed with 1 mL of PBS and centrifuged at 200 x g for 5 minutes. GMR-AML1 cells were suspended in 100 µL of 0.5% BSA/PBS, placed on ice, and the expression of cell surface markers was determined by flow cytometry. Percent expression of each cell surface marker is reported relative to the respective IgG isotype control.

**Immunohistochemistry analysis of GMR-AML1 cells.** H&E staining was performed on 4 µm-thick formalin-fixed paraffin-embedded (FFPE) tissue section of GMR-AML1 cells cut using a microtome in the Hematopathology department at MD Anderson Cancer Center, Houston TX. De-paraffinization and staining were performed as described previously*^26, 27^*.

**FISH analysis.** Fluorescence in situ hybridization (FISH) was performed as described previously*^28^*. An LSI *MYC* or KMT2A dual‐color break apart probe set (Abbott Laboratories) was used to assess rearrangements involving the *MYC* locus on chromosome 8q24 or KMT2A locus on chromosome 11q23. Signals from at least 200 nuclei were analyzed.

**Karyotyping analysis.** Cytogenetic analysis was performed on G-banded metaphase cells prepared from unstimulated 24-hour and 48-hour cultured samples as described previously*^29^*. A total of 20 metaphases (10 from each culture) were analyzed.

**Assessment of apoptosis by annexin-V staining.** Untreated control or drug-treated GMR-AML1 cells were stained with Annexin-V (Pharmingen, San Diego, CA) and TO-PRO-3 iodide (Life Technologies, Carlsbad, CA) and the percentages of apoptotic cells were determined by flow cytometry after 48 hours.

**Assessment of percentage non-viable cells.** Following designated treatments, GMR-AML1 or PD FPD or FPD-MM cells were stained with propidium iodide or TO-PRO-3 iodide (Life Technologies, Carlsbad, CA) and analyzed by flow cytometry on a BD Accuri CFlow-6 flow cytometer (BD Biosciences, San Jose, CA). To analyze synergism, cells were treated with combinations for 96 hours and the percentages of TO-PRO-3 iodide-positive, non-viable cells were determined by flow cytometry. We utilized matrix dosing of agents in combinations to allow synergy assessment by Delta Synergy scoring utilizing the SynergyFinder online web application tool (<http://synergyfinder.fimm.fi/>)*^30-32^*.

**Protein synthesis assay.** The quantification of nascent polypeptide elongation inhibition was performed as described by the manufacture (ab239725 Abcam; Cambridge, MA). In brief, GMR-AML1 cells were treated with mebendazole, volasertib, alisertib, or adavosertib for 16 hrs. In the last 30 minutes of treatment, cells were incubated in the presence of OP-Puromycin (OPP) which is incorporated into nascent polypeptide chains. The cells were then fixed and permeabilized. Next, via click chemistry, OPP was subsequently labeled with fluorescent azide and inhibition of protein synthesis was quantified by flow cytometry.

**RNA isolation and quantitative polymerase chain reaction.** Following the designated treatments, total RNA was isolated from AML cells utilizing a PureLink RNA Mini kit from Ambion, Inc. (Austin, TX) and reverse transcribed with a High Capacity Reverse Transcription kit from Life Technologies (Carlsbad, CA). Quantitative real-time PCR analysis for the expression of target genes was performed on cDNA using TaqMan probes and a TaqMan Universal PCR Mastermix from Applied Biosystems (Foster City, CA). Relative mRNA expression was normalized to the expression of GAPDH and compared to the untreated cells.

**Cell lysis and protein quantitation.** Knockout**,** untreated, or drug-treated cells were centrifuged, and the cell pellets were incubated in lysis buffer on ice for 20 minutes*^33^*. After centrifugation, an aliquot of each cell lysate was diluted 1:10 and the protein content was quantitated using a BCA protein quantitation kit (Pierce, Rockford, IL), according to the manufacturer’s protocol. Protein concentrations were determined by comparing the absorbance at 562 nm compared to a known concentration range of bovine serum albumin (BSA) from 0.125 mg to 2 mg/mL.

**SDS-PAGE and immunoblot analyses.** Thirty micrograms of total cell lysate were used for SDS-PAGE. Western blot analyses were performed on total cell lysates using specific antisera or monoclonal antibodies. Blots were washed with 1× PBST, then incubated in IRDye 680RD goat anti-mouse (RRID:AB_10956588) or IRDye 800CW goat anti-rabbit (RRID:AB_621843) secondary antibodies (LI-COR, Lincoln, NE) for 1 h, washed three times in 1× Phosphate Buffered Saline with Tween®20 (PBST) and scanned with an Odyssey CLX Infrared Imaging System utilizing Image Studio 5.0 Software (RRID:SCR_015795) (LI-COR, Lincoln, NE). The expression levels of β-Actin or GAPDH in the cell lysates were used as the loading control for the western blots. Immunoblot analyses were performed at least twice. Representative immunoblots were subjected to densitometry analysis. Densitometry analysis was performed using ImageJ software*^34^*.

**Single cell next-generation mass cytometry ‘CyTOF’ analysis of primary FPD-MM cells.** Primary, patient-derived FPD-MM cells were treated with 100 nM of homoharringtonine for 16 hrs or 1,000 nM of mebendazole for 24 hours. At the end of treatment, cells were blocked with staining buffer (0.5% BSA/PBS) for 30 minutes, then a cocktail of extracellular antibodies (CLEC12A [CLL-1], CD123, CD117, CD244, CD86, and CD11b, and CD34) conjugated to transition element isotopes were added and incubated for 1 hour at room temperature (RT). For viability staining, a 5 µM concentration of cisplatin was added and incubated at RT for 2 minutes. Cells were washed with staining buffer, centrifuged at 500 x g for 5 minutes and staining buffer was vacuum aspirated. Cells were fixed with 100 µL of 1.6% paraformaldehyde (PFA) for 10 minutes at room temperature. Following this, cells were permeabilized with 900 µL of ice-cold 100% methanol (90% volume) at -20°C for at least 20 minutes. Next, cells were washed with 1 ml of staining buffer to remove the paraformaldehyde/methanol solution. Cells were blocked in 50 µL of staining buffer for 30 minutes and a cocktail of intracellular antibodies (CDK6, Runx1, pRb, p53, PU.1, Bfl1, Mcl1, EVI1, c-Myc, MPO1, Bax, clPARP, Hoxa9, Meis1, and Bcl2) conjugated to transition element isotopes was added to be used as tags in atomic mass spectrometric analysis of the cells. Cells were incubated for 1 hour at room temperature, then washed with staining buffer at 500 x g for 5 minutes. Intercalator was added (500 µL of 1:1000 Ir-intercalator diluted in 1.6% PFA/1X PBS) and cells were incubated overnight at 4°C. Cells were washed twice (500 x g for 5 minutes per wash) in staining buffer, then counted using a Countess II counting device. Following the last wash, 1 x 10^6^ cells were suspended in 100 µL of de-ionized water and incubated overnight at 4°C. Time-of-flight mass spectrometry (CyTOF) measured multiple different cellular parameters simultaneously in each cell. The absolute fold-change of protein expression changes over control cells within the CLEC12A [CLL-1] Hi, CD123 Hi, CD117 Hi, CD244 Lo, CD86 Lo, and CD11b Lo population was analyzed by the Astrolabe Cytometry Platform (Astrolabe, Fort Lee, NJ).

**Reverse phase protein array (RPPA) analysis.** Mebendazole inhibitor-treated cells were treated in biologic triplicates for 16 hours. At the end of treatment, cells were harvested, washed once with 1X PBS and snap frozen in liquid nitrogen. RPPA analysis was performed in the Functional Proteomics RPPA core facility at the MD Anderson Cancer Center. This array allows the simultaneous detection of 494 unique antibodies against human proteins. This array is curated and highly validated. Briefly, cell lysates were serially diluted two-fold for 5 dilutions (from undiluted to 1:16 dilution) and arrayed on nitrocellulose-coated slides in an 11 x 11 format. Samples were probed with antibodies by tyramide-based signal amplification approach and visualized by DAB colorimetric reaction. Slides were scanned on a flatbed scanner to produce 16-bit tiff image. Spots from tiff images were identified and the density was quantified by Array-Pro Analyzer. Relative protein expression for each sample were normalized by interpolation of each dilution curves from the "standard curve" (supercurve) of the slide (antibody). Supercurve is constructed by a script in R, written by the Bioinformatics Department at the University of Texas MD Anderson Cancer Center*^35^*. These values (given as Log2 values) are defined as Supercurve Log2 (Raw) values and imported into an Excel worksheet. All the data points were normalized for protein loading and transformed to linear value, designated as "Normalized Linear" (labeled "NormLinear" in the worksheet). "Normalized Linear" values were transformed to Log2 values (labeled "NormLog2" in worksheet), and then median-centered for hierarchical clustering analysis (labeled "NormLog2_MedianCentered" in the worksheet). Median-centered values were then formatted for heatmap generation in the "Format for Heatmap" worksheet. Our data were further processed and our heatmaps display only proteins that were altered greater than or equal to 25% up or down and had a p-value of less than 0.05. Multiple hypotheses testing correction was applied using the false discovery rate (fdr) method as implemented in the R statistical system.

**In vivo model of FPD-MM.** All in vivo studies were approved by and conducted in accordance with the guidelines of the IACUC at the M.D. Anderson Cancer Center, an AAALAC-accredited facility. Female NOD.Cg-Prkdc^scid^ Il2rg^tm1Wjl^/SzJ (NSG) mice (stock number: 005557; 4-6 weeks of age) [Jackson Labs, Bar Harbor, ME; RRID: IMSR_JAX:005557] were exposed to 2.5 Gy of radiation. The following day, mice were injected in the lateral tail vein. Mice were imaged utilizing a Xenogen Lumina in vivo imaging system to document engraftment before treatment was initiated. Mice in all models described below were randomized into groups based on equivalent mean bioluminescent intensity to control for variation in cell engraftment and variation between different treatment groups. For the first mouse model, mice (n=10 per cohort) were injected in the lateral tail vein with 1 x 10^6^ GFP-luciferase expressing GMR-AML1 cells and monitored for five days. Treatment was initiated on day six. The first cohort of mice were treated with 1.0 mg/kg omacetaxine mepesuccinate daily for three weeks, followed by one week at 0.5 mg/kg via subcutaneous injection. The second cohort of mice were treated with 20 mg/kg of mebendazole daily for four weeks by oral gavage. The final cohort of mice were treated with 40 mg/kg of mebendazole for three weeks and then reduced to 20 mg/kg for one week by oral gavage. For the second mouse model, mice (n=10 per cohort) were injected in the lateral tail vein with 1 x 10^6^ GFP-luciferase expressing GMR-AML1 cells and monitored for five days. Treatment was initiated on day six. The first cohort of mice were treated with 15 mg/kg of volasertib one time per week for two weeks, followed by a one-week holiday. Then, the mice were treated for three weeks at 7.5 mg/kg by oral gavage. The second cohort of mice were treated with 20 mg/kg of mebendazole daily for six weeks by oral gavage. The final cohort of mice were treated with volasertib and mebendazole at the dose and schedule indicated above. All mice in each treatment cohort were imaged utilizing a Xenogen Lumina in vivo imaging system once per week to monitor disease status and treatment efficacy. Total bioluminescence was recorded as photons/second.

**Power analysis for in vivo studies**. With a sample size of 10 mice per group, we can achieve 79.5% power to detect a difference of overall survival at a significance level of 0.05 with one-sided log-rank test, assuming 30% of mouse-survival at the end of study in the experimental group.

**Statistical analysis**. Significant differences between values obtained in AML cells treated with different experimental conditions compared to untreated control cells were determined using the Student’s t-test in GraphPad V9. For the *in vivo* mouse models, a two-tailed, unpaired t-test was utilized for comparing total bioluminescent flux. For survival analysis, a Kaplan-Meier plot and a Mantel–Cox log rank test were utilized for comparisons of different cohorts. P values of < 0.05 were assigned significance.

**Data and Software availability**. RNA-Seq and ChIP-Seq datasets have been deposited in GEO and assigned accession IDs.

**REFERENCES for Methods**

[1] Sanger, F., Nicklen, S., and Coulson, A. R. (1977) DNA sequencing with chain-terminating inhibitors, *Proc Natl Acad Sci U S A* *74*, 5463-5467.

[2] Khan, M., Cortes, J., Kadia, T., Naqvi, K., Brandt, M., Pierce, S., Patel, K. P., Borthakur, G., Ravandi, F., Konopleva, M., Kornblau, S., Kantarjian, H., Bhalla, K., and DiNardo, C. D. (2017) Clinical Outcomes and Co-Occurring Mutations in Patients with RUNX1-Mutated Acute Myeloid Leukemia, *Int J Mol Sci* *18*.

[3] Schmidl, C., Rendeiro, A. F., Sheffield, N. C., and Bock, C. (2015) ChIPmentation: fast, robust, low-input ChIP-seq for histones and transcription factors, *Nat Methods* *12*, 963-965.

[4] Shen, L., Shao, N. Y., Liu, X., Maze, I., Feng, J., and Nestler, E. J. (2013) diffReps: detecting differential chromatin modification sites from ChIP-seq data with biological replicates, *PLoS One* *8*, e65598.

[5] Robinson, J. T., Thorvaldsdottir, H., Winckler, W., Guttman, M., Lander, E. S., Getz, G., and Mesirov, J. P. (2011) Integrative genomics viewer, *Nat Biotechnol* *29*, 24-26.

[6] Thorvaldsdottir, H., Robinson, J. T., and Mesirov, J. P. (2013) Integrative Genomics Viewer (IGV): high-performance genomics data visualization and exploration, *Brief Bioinform* *14*, 178-192.

[7] Loven, J., Hoke, H. A., Lin, C. Y., Lau, A., Orlando, D. A., Vakoc, C. R., Bradner, J. E., Lee, T. I., and Young, R. A. (2013) Selective inhibition of tumor oncogenes by disruption of super-enhancers, *Cell* *153*, 320-334.

[8] Dobin, A., Davis, C. A., Schlesinger, F., Drenkow, J., Zaleski, C., Jha, S., Batut, P., Chaisson, M., and Gingeras, T. R. (2013) STAR: ultrafast universal RNA-seq aligner, *Bioinformatics* *29*, 15-21.

[9] Li, H., Handsaker, B., Wysoker, A., Fennell, T., Ruan, J., Homer, N., Marth, G., Abecasis, G., Durbin, R., and Genome Project Data Processing, S. (2009) The Sequence Alignment/Map format and SAMtools, *Bioinformatics* *25*, 2078-2079.

[10] Love, M. I., Huber, W., and Anders, S. (2014) Moderated estimation of fold change and dispersion for RNA-seq data with DESeq2, *Genome Biol* *15*, 550.

[11] Benjamini, Y., and Hochberg, Y. (1995) Controlling the False Discovery Rate: A Practical and Powerful Approach to Multiple Testing, *Journal of the Royal Statistical Society: Series B (Methodological)* *57*, 289-300.

[12] Subramanian, A., Tamayo, P., Mootha, V. K., Mukherjee, S., Ebert, B. L., Gillette, M. A., Paulovich, A., Pomeroy, S. L., Golub, T. R., Lander, E. S., and Mesirov, J. P. (2005) Gene set enrichment analysis: a knowledge-based approach for interpreting genome-wide expression profiles, *Proc Natl Acad Sci U S A* *102*, 15545-15550.

[13] Liberzon, A., Subramanian, A., Pinchback, R., Thorvaldsdottir, H., Tamayo, P., and Mesirov, J. P. (2011) Molecular signatures database (MSigDB) 3.0, *Bioinformatics* *27*, 1739-1740.

[14] Coarfa, C., Grimm, S. L., Katz, T., Zhang, Y., Jangid, R. K., Walker, C. L., Moorthy, B., and Lingappan, K. (2020) Epigenetic response to hyperoxia in the neonatal lung is sexually dimorphic, *Redox Biol* *37*, 101718.

[15] Krzywinski, M., Schein, J., Birol, I., Connors, J., Gascoyne, R., Horsman, D., Jones, S. J., and Marra, M. A. (2009) Circos: an information aesthetic for comparative genomics, *Genome Res* *19*, 1639-1645.

[16] Korsunsky, I., Millard, N., Fan, J., Slowikowski, K., Zhang, F., Wei, K., Baglaenko, Y., Brenner, M., Loh, P. R., and Raychaudhuri, S. (2019) Fast, sensitive and accurate integration of single-cell data with Harmony, *Nat Methods* *16*, 1289-1296.

[17] Stuart, T., Butler, A., Hoffman, P., Hafemeister, C., Papalexi, E., Mauck, W. M., 3rd, Hao, Y., Stoeckius, M., Smibert, P., and Satija, R. (2019) Comprehensive Integration of Single-Cell Data, *Cell* *177*, 1888-1902 e1821.

[18] Becht, E., McInnes, L., Healy, J., Dutertre, C. A., Kwok, I. W. H., Ng, L. G., Ginhoux, F., and Newell, E. W. (2018) Dimensionality reduction for visualizing single-cell data using UMAP, *Nat Biotechnol*.

[19] Hanzelmann, S., Castelo, R., and Guinney, J. (2013) GSVA: gene set variation analysis for microarray and RNA-seq data, *BMC Bioinformatics* *14*, 7.

[20] Stuart, T., Srivastava, A., Madad, S., Lareau, C. A., and Satija, R. (2021) Single-cell chromatin state analysis with Signac, *Nat Methods* *18*, 1333-1341.

[21] Feng, J., Liu, T., Qin, B., Zhang, Y., and Liu, X. S. (2012) Identifying ChIP-seq enrichment using MACS, *Nat Protoc* *7*, 1728-1740.

[22] Heinz, S., Benner, C., Spann, N., Bertolino, E., Lin, Y. C., Laslo, P., Cheng, J. X., Murre, C., Singh, H., and Glass, C. K. (2010) Simple combinations of lineage-determining transcription factors prime cis-regulatory elements required for macrophage and B cell identities, *Mol Cell* *38*, 576-589.

[23] Ramirez, F., Ryan, D. P., Gruning, B., Bhardwaj, V., Kilpert, F., Richter, A. S., Heyne, S., Dundar, F., and Manke, T. (2016) deepTools2: a next generation web server for deep-sequencing data analysis, *Nucleic Acids Res* *44*, W160-165.

[24] Kent, W. J., Sugnet, C. W., Furey, T. S., Roskin, K. M., Pringle, T. H., Zahler, A. M., and Haussler, D. (2002) The human genome browser at UCSC, *Genome Res* *12*, 996-1006.

[25] Labun, K., Montague, T. G., Gagnon, J. A., Thyme, S. B., and Valen, E. (2016) CHOPCHOP v2: a web tool for the next generation of CRISPR genome engineering, *Nucleic Acids Res* *44*, W272-276.

[26] Khoury, J. D., Wang, W. L., Prieto, V. G., Medeiros, L. J., Kalhor, N., Hameed, M., Broaddus, R., and Hamilton, S. R. (2018) Validation of Immunohistochemical Assays for Integral Biomarkers in the NCI-MATCH EAY131 Clinical Trial, *Clin Cancer Res* *24*, 521-531.

[27] Sukswai, N., and Khoury, J. D. (2019) Immunohistochemistry Innovations for Diagnosis and Tissue-Based Biomarker Detection, *Curr Hematol Malig Rep* *14*, 368-375.

[28] Tang, Z., Li, Y., Wang, W., Yin, C. C., Tang, G., Aung, P. P., Hu, S., Lu, X., Toruner, G. A., Medeiros, L. J., and Khoury, J. D. (2018) Genomic aberrations involving 12p/ETV6 are highly prevalent in blastic plasmacytoid dendritic cell neoplasms and might represent early clonal events, *Leuk Res* *73*, 86-94.

[29] Khoury, J. D., Sen, F., Abruzzo, L. V., Hayes, K., Glassman, A., and Medeiros, L. J. (2003) Cytogenetic findings in blastoid mantle cell lymphoma, *Hum Pathol* *34*, 1022-1029.

[30] Ianevski, A., Giri, A. K., and Aittokallio, T. (2020) SynergyFinder 2.0: visual analytics of multi-drug combination synergies, *Nucleic Acids Res* *48*, W488-W493.

[31] Ianevski, A., He, L., Aittokallio, T., and Tang, J. (2020) SynergyFinder: a web application for analyzing drug combination dose-response matrix data, *Bioinformatics* *36*, 2645.

[32] Ianevski, A., Giri, A. K., and Aittokallio, T. (2022) SynergyFinder 3.0: an interactive analysis and consensus interpretation of multi-drug synergies across multiple samples, *Nucleic Acids Res*.

[33] Fiskus, W., Verstovsek, S., Manshouri, T., Rao, R., Balusu, R., Venkannagari, S., Rao, N. N., Ha, K., Smith, J. E., Hembruff, S. L., Abhyankar, S., McGuirk, J., and Bhalla, K. N. (2011) Heat shock protein 90 inhibitor is synergistic with JAK2 inhibitor and overcomes resistance to JAK2-TKI in human myeloproliferative neoplasm cells, *Clin Cancer Res* *17*, 7347-7358.

[34] Schneider, C. A., Rasband, W. S., and Eliceiri, K. W. (2012) NIH Image to ImageJ: 25 years of image analysis, *Nat Methods* *9*, 671-675.

[35] Troncale, S., Barbet, A., Coulibaly, L., Henry, E., He, B., Barillot, E., Dubois, T., Hupe, P., and de Koning, L. (2012) NormaCurve: a SuperCurve-based method that simultaneously quantifies and normalizes reverse phase protein array data, *PLoS One* *7*, e38686.
